# Common Pathogenic Mechanisms in Centronuclear and Myotubular Myopathies and Latest Treatment Advances

**DOI:** 10.3390/ijms222111377

**Published:** 2021-10-21

**Authors:** Raquel Gómez-Oca, Belinda S. Cowling, Jocelyn Laporte

**Affiliations:** 1 Department of Translational Medicine and Neurogenetics, Institut de Génétique et de Biologie Moléculaire et Cellulaire (IGBMC), 67400 Illkirch, France; gomezocr@igbmc.fr; 2 Institut National de la Santé et de la Recherche Médicale (INSERM), U1258, 67400 Illkirch, France; 3 Centre National de la Recherche Scientifique (CNRS), UMR7104, 67400 Illkirch, France; 4 Strasbourg University, 67081 Strasbourg, France; 5 Dynacure, 67400 Illkirch, France; belinda.cowling@dynacure.com

**Keywords:** centronuclear myopathy, myotubular myopathy, myotubularin, dynamin, amphiphysin, ryanodine receptor, autophagy, triads, membrane trafficking, satellite cell

## Abstract

Centronuclear myopathies (CNM) are rare congenital disorders characterized by muscle weakness and structural defects including fiber hypotrophy and organelle mispositioning. The main CNM forms are caused by mutations in: the *MTM1* gene encoding the phosphoinositide phosphatase myotubularin (myotubular myopathy), the *DNM2* gene encoding the mechanoenzyme dynamin 2, the *BIN1* gene encoding the membrane curvature sensing amphiphysin 2, and the *RYR1* gene encoding the skeletal muscle calcium release channel/ryanodine receptor. MTM1, BIN1, and DNM2 proteins are involved in membrane remodeling and trafficking, while RyR1 directly regulates excitation-contraction coupling (ECC). Several CNM animal models have been generated or identified, which confirm shared pathological anomalies in T-tubule remodeling, ECC, organelle mispositioning, protein homeostasis, neuromuscular junction, and muscle regeneration. Dynamin 2 plays a crucial role in CNM physiopathology and has been validated as a common therapeutic target for three CNM forms. Indeed, the promising results in preclinical models set up the basis for ongoing clinical trials. Another two clinical trials to treat myotubular myopathy by MTM1 gene therapy or tamoxifen repurposing are also ongoing. Here, we review the contribution of the different CNM models to understanding physiopathology and therapy development with a focus on the commonly dysregulated pathways and current therapeutic targets.

## 1. Introduction

Centronuclear myopathies (CNM) are a subgroup of congenital muscle disorders encompassing myotubular myopathy and are characterized by the abnormal nuclei position in the center of the myofibers in the absence of excessive regeneration, unlike what is commonly observed in dystrophies [1]. The first published patient description was in 1996 by Spiro et al. [2] and since then, major advances have been made in the identification of causative genes. Clinical presentation and inheritance are heterogenous and can be classified into three main groups: the most severe X-linked CNM (XLMTM, myotubular myopathy) caused by mutations in *MTM1* encoding myotubularin (MTM1) [3]; autosomal dominant CNM (ADCNM) caused by dominant mutations in *DNM2* encoding dynamin 2 (DNM2) [4] or in *BIN1* encoding amphiphysin 2 (BIN1) [5]; and autosomal recessive CNM (ARCNM) caused by a mutation in *BIN1* [6] or *RYR1* encoding ryanodine receptor (RyR1) [7,8]. Around 16% of CNM cases have an unknown genetic cause [9]. Mutations in other genes including *TTN* [10], *SPEG* [11], *CACNA1S* [12], and *ZAK* [13] have been identified in congenital myopathies with a CNM-like phenotype combined with other features. In the present review we aim to provide an overview of the clinical and pathological aspects of CNM with a focus on the main genetic forms with mutations in *MTM1*, *BIN1*, *DNM2*, and *RYR1* genes, and highlight common disease mechanisms. Finally, updates on novel therapeutic targets and a current translation of different treatment approaches to human clinical trials will be discussed.

## 2. MTM1 and Myotubular Myopathy

### 2.1. Disease Presentation and Genetics

X-linked centronuclear myopathy or myotubular myopathy (XLCNM, XLMTM, OMIM#310400) is caused by recessive mutations in the *MTM1* gene encoding the 3′-phosphoinositide phosphatase myotubularin [3] (Table 1). The disease name comes from the resemblance of patient muscle fibers to fetal myotubes upon the analysis of muscle biopsies [2]. More than 300 loss-of-function mutations have been identified without specific hotspots [14,15,16,17,18], most resulting in a strong decrease in MTM1 protein levels [19,20].

XLMTM is a rare congenital myopathy that affects about 1/50,000 newborn males per year [21]. Recent estimations have proposed that it accounts for the largest proportion of CNM patients, representing around 57% of the prevalent population [9]. It is characterized by a severe neonatal or prenatal myopathy, constituting one of the most severe forms of CNM. It has a poor prognosis as 60% of patients died within the first two years of life, while some affected boys survived into childhood and even adulthood [14].

The general clinical picture is composed of severe hypotonia, muscle atrophy, and generalized weakness at birth. Most patients also present respiratory insufficiency, requiring immediate ventilatory assistance, and swallowing difficulties, needing a feeding tube. Moderate ptosis (drooping of the upper eyelid), ophthalmoplegia (weakness of eye muscles), and scoliosis are often observed [21,22]. Non-muscle phenotypes have been frequently reported. It is frequent to observe dolichocephaly (large head relative to its width). Pyloric stenosis and cavernous hemangiomas of the liver were reported as complications in long-term survivors [23]. Following the fatal death of three patients in the ASPIRO clinical study (MTM1 gene therapy, see therapy section) possibly linked to liver complications, hepatobiliary disease in XLMTM was further characterized in two recent studies [24,25]. Overall, it was shown that hepatobiliary disease, and in particular, cholestasis, is a common comorbidity in XLMTM patients that should be closely monitored. It can manifest from an early age, especially after infection or vaccination, and presents with an increase in liver enzymes (transaminases and bilirubin) and liver ultrasound abnormalities as hepatic peliosis or gallstones. It was not correlating clinical severity nor *MTM1* mutation.

Most female XLMTM carriers were first assumed to be asymptomatic, but recent studies have shown they can have symptoms with a wide spectrum of clinical involvement from severe neonatal and generalized weakness with respiratory dysfunction to more commonly observed milder forms in adulthood [26,27]. Prevalence of manifesting XLMTM carriers may be higher than currently assumed as demonstrated with a new cohort of 76 female carriers published in 2021 with 51% manifesting carriers [28]. It is characteristic to observe asymmetric weakness and growth, facial involvement, ptosis, and skeletal and joint abnormalities [26,27]. The differences in severities are not explained by the *MTM1* mutation. Skewed X-chromosome inactivation (XCI) was noted in some female carriers (six patients out of 32 with a ratio of 95:5) [26], although this was demonstrated to not be correlated with the severity of the phenotypes [27]. Severity in females may be explained by disease modifier genes or the susceptibility of the *MTM1* gene to escape XCI.

Main morphological features observed in muscle biopsies from patients with XLMTM are smaller round myofibers with centralized nuclei, predominance of type 1 slowly contracting fibers and lack of oxidative activity in the periphery of the fibers. Centralized nuclei are surrounded by an area depleted of myofilaments containing mitochondria, glycogen, and some tubular structures [1]. Necklace fibers have been reported in late onset XLMTM cases [30] and female carriers [26]. Patients were mildly affected during childhood and worsening after the first and second decades of life. Necklace are basophilic rings located underneath the sarcolemma following the contour of the cell and composed of glycogen, mitochondria, and reticulum as observed with periodic acid–Schiff (PAS), oxidative staining, and electron microscopy. In those patients, myonuclei were usually aligned in the necklace.

### 2.2. Myotubularin Function and Animal Models for XLMTM

#### 2.2.1. Myotubularin

**Figure 1:**
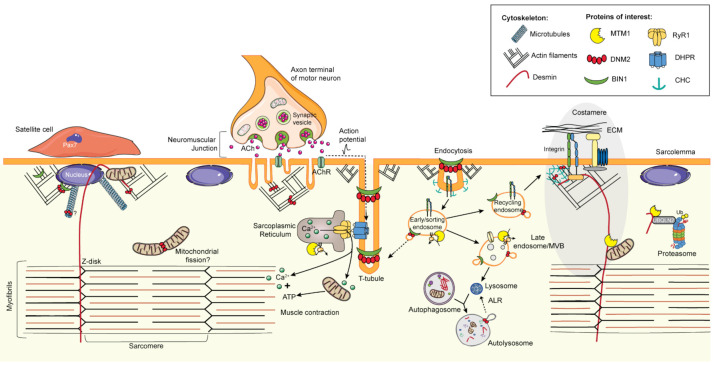
Schematic illustration of localization and functions of MTM1, DNM2, BIN1, and RyR1 in skeletal muscle. ECM (extracellular matrix), ALR (autophagic lysosome reformation), Ub (ubiquitin), Ach (acetylcholine), MVB (multivesicular bodies). The figure uses modified images from Servier Medical Art Commons Attribution 3.0 Unported License (http://smart.servier.com, accessed on 29 August 2021).

Myotubularin (MTM1) is a ubiquitously expressed phosphosinositide (PI) phosphatase that regulates intracellular vesicle trafficking through the spatial and temporal regulation of PI phosphorylation. PIs are lipids that determine membrane identity and regulate protein recruitment [45,46]. MTM1 specifically dephosphorylates position 3 of phosphatidylinositol-3,5-bisphosphate (PI(3,5)P_2_ to PI(5)P) and phosphatidylinositol-3-phosphate (PI(3)P to PI), second messenger lipids produced by PI 3-kinase (PI3K) and 5-kinase (PI5K), respectively [47,48,49]. These lipids are important to regulate intracellular processes such as autophagy or vesicle sorting within and through endosomal compartments [46]. MTM1 is part of a highly conserved protein family conserved throughout evolution in Eucaryotes [50,51] (Figure 1).

**Figure 2:**
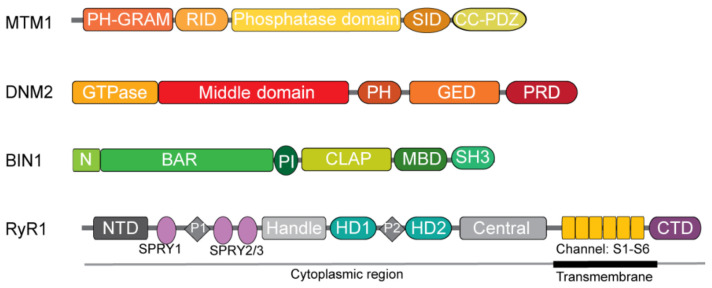
Scheme of MTM1, DNM2, BIN1, and RyR1 protein domains. MTM1: PH-GRAM (Pleckstrin homology-glucosyltransferase; Rab-like GTPase activator and myotubularin), RID (Rac1-induced recruitment domain), SID (SET interacting domain), CC-PDZ (coiled coil; PSD95, disc large, ZO-1). DNM2: PH (Pleckstrin-homology), GED (GTPase effector domain), PRD (proline-rich domain). BIN1: N (N-terminal amphipathic helix α), BAR (Bin-amphiphysin-Rvs), PI (phosphoinositides), CLAP (clathrin and AP2 binding), MBD (Myc binding domain), SH3 (SRC homology 3). RyR1: NTD (N-terminal domain), SPRY (SPla and the RYanodine receptor), HD (helical domain), CTD (C-terminal domain).

The MTM1 protein has the following protein domains (Figure 2), from the N-terminal to C-terminal [51]. The PH-GRAM domain (Pleckstrin homology-glucosyltransferase, Rab-like GTPase activator and myotubularin) can bind lipids, especially PI(3,5)P_2_, which is enriched in early endosomes [52], and may also interact with effector proteins. The RID domain (Rac1-induced recruitment domain or myotubularin-related domain) regulates its recruitment to the plasma membrane [53] and interacts with the muscle intermediate filament desmin [31]. The phosphatase domain encompasses a signature motif conserved among phosphatases and is responsible for phosphoinositide dephosphorylation [48]. The SID (SET interacting domain) that interacts with SET domain proteins involved in epigenetic regulation [54]. A coiled coil domain is essential for homodimerization and/or heterodimerization of myotubularin. Finally, a PDZ (PSD95, disc large, ZO-1) binding site is localized at the C-terminal, mediating protein–protein interactions.

Mutations in *MTM1* spread throughout all the different domains with no hotspots and no clear genotype–phenotype correlations [55,56,57].

#### 2.2.2. Mouse Models for XLMTM

The development of animal models mimicking XLMTM have been essential to decipher the pathomechanisms and validate several therapeutic targets. Here, we focus on animal models for which specific orthologs of human MTM1 are found (mammals and fish) (Table 2).

A first XLMTM mouse model was generated through exon 4 deletion (*Mtm1*δ4 line, *Mtm1* knockout *Mtm1*^−/y^), leading to complete knockout of the MTM1 protein in all tissues [58,59]. *Mtm1*^−/y^ mice have a reduced lifespan of 1–3 months, probably dying due to respiratory failure. Males develop a progressive and generalized myopathy with severe muscle weakness and decrease in body weight starting at 3–4 weeks of age. No phenotype has been reported to date in heterozygous female mice (*Mtm1^+/−^*). Conditional ablation of this exon only in skeletal muscle using a Cre recombinase expression driven by the HSA (human skeletal actin) promotor causes almost identical phenotypes, pointing to myotubularin loss in muscle as the main cause of the disease. Histopathological hallmarks in muscle biopsies resemble those observed in patients with high incidence of small fibers with centralized or internalized nuclei, and a peripheral halo depleted of oxidative activity underlying mitochondria mispositioning. The fact that mice, unlike patients, are not affected from birth can be explained due to the different timing of late myogenesis as muscle development is completed in human before birth but finalizes at two weeks of age in mice. Ultrastructural analysis showed Z-line misalignment, decreased number of triads, swollen sarcoplasmic reticulum, and accumulation of mitochondria and glycogen around centralized nuclei, as reported in XLMTM patients. These results point to myotubularin as a key player for muscle skeletal maintenance.

A *Mtm1* knock-in (KI) mouse model was generated carrying the R69C mutation (*Mtm1^R69C/y^)* previously identified in patients with a mild phenotype [60]. Analysis of this model revealed that this mutation causes the skipping of exon 4, leading to premature termination of myotubularin translation. However, some full-length *Mtm1* mRNA with the missense mutation is expressed, resulting in a milder phenotype compared to *Mtm1*^−/y^ mice with a median survival of 66 weeks. Muscle weakness appears at eight weeks of age, but does not progress with age, with muscle fiber hypotrophy and nuclei centralization already present at four weeks. Interestingly, the central nuclei increase over time, but do not correlate with worsening of muscle force [60].

A third *Mtm1* mouse model was developed by a gene trap strategy (*Mtm1^gt^*^/*y*^) where a vector with the neomycin reporter was inserted into intron 1 of the *Mtm1* gene, disrupting its expression [61]. *Mtm1^gt^*^/*y*^ mice survive for at least six weeks and develop a progressive myopathy from three weeks with histological defects as internalize nuclei, decreased myofiber size, and disorganized sarcomeres, similar to the *Mtm1*^−/y^ mouse.

Recently, novel *Mtm1* knockout mouse models were generated using CRISPR technology by deletion of the 5-base pair (bp) (*Mtm1*^Δ5/y^) or 7-bp (*Mtm1*^Δ7/y^) in the exon 3 [62]. As in *Mtm1*^−/y^ mice, these mutant mice have a maximum lifespan of eight weeks, smaller body weight than the controls, and histopathological hallmarks similar to XLMTM patients [62].

#### 2.2.3. Other Animal Models for XLMTM

Zebrafish: The morpholino antisense technology was used in zebrafish to reduce the level of myotubularin (*mtm1* morphant) and resulted in impairment of motor function and histopathologic changes resembling those seen in patients with mispositioned nuclei, fiber hypotrophy, and abnormal triads [34] (Table 2). Similar phenotypes were found in a zebrafish mutant created using zinc finger nucleases to produce a deletion of 8-bp in exon 5 of the *mtm1* gene (*mtm1*-null), leading to frameshift and early stop mutation [63].

Dogs: In addition, two spontaneous canine models have been identified for XLMTM (Table 2). First, a Labrador retriever dog harboring the mutation N155K (exon 7) in the *MTM1* gene [64], followed by the identification of the mutation Q384P (exon 11) in a Rottweiler dog [65]. The affected dogs developed a progressive myopathy with generalized muscle weakness and atrophy, and mispositioned nuclei and mitochondria together with type 1 fiber predominancy in muscle biopsies.

## 3. DNM2 and Autosomal Dominant Centronuclear Myopathy

### 3.1. Disease Presentation and Genetics

DNM2-related autosomal dominant centronuclear myopathy (DNM2-related ADCNM, OMIM#160150) is caused by heterozygous mutations in *DNM2*, encoding the large ubiquitous GTPase dynamin 2 (Table 1) [4]. More than 15 different CNM mutations affecting mainly the stalk and PH domains (see below) have been reported [76]. They are suspected to be gain-of-function mutations that result in more stable oligomer structures and increased GTPase activity in vitro compared to wild-type dynamin 2 [77,78,79]. Different heterozygous *DNM2* mutations have been found in patients with dominant intermediate and axonal forms of Charcot Marie Tooth neuropathy (CMTDIB and CMT2B OMIM#606482) affecting peripheral nerves. Of note, a homozygous mutation was also reported in a single family with lethal congenital contracture syndrome (OMIM#615368). In this review, we will only focus on *DNM2* mutations causing CNM.

CNM, caused by *DNM2* mutations, accounts for around 12% of CNM patients. The yearly incidence of DNM2-related ADCNM was estimated to be 21 newborns globally (including Europe, the United States, Japan, and Australia) per year [9]. It presents a large clinical variability ranging from severe and neonatal onset to milder forms, with adult onset [4,37,38] DNM2-related ADCNM patients generally having a better prognosis even in neonatal cases, in comparison with XLMTM.

Adult or adolescent cases present with delayed motor milestones during childhood, in particular, for walking and climbing stairs or running [22]. Severe pediatric cases, however, often present generalized muscle weakness and hypotonia together with moderate facial weakness, ophthalmoplegia, and ptosis. Most patients with early onset-severe form develop breathing complications at the end of the first decade [76]. Common to all DNM2 cases is hyporeflexia or areflexia [76]. Achilles tendon contractures are frequently reported for *DNM2* patients [80]. Coexistence of typical CNM-features with peripheral neuropathy with mild axonal peripheral nerve involvement has been described in patients carrying a CNM mutation [81,82].

The analysis of muscle biopsies of DNM2-related ADCNM shows a significantly high proportion of muscle fibers with central and internal nuclei, predominance of type 1 fibers, and fiber size heterogeneity. The central internuclear space is depleted of sarcomeres and occupied by glycogen granules, mitochondria, endoplasmic reticulum, and Golgi apparatus [1]. As a discriminatory feature from other CNM forms, myofibers present characteristic radiating sarcoplasmic strands (RSS, also named “spokes of a wheel”), visible with oxidative reactions and easily observed in electron microscopy with a radial distribution of the intermyofibrillar sarcoplasmic reticulum around the centralized nuclei.

### 3.2. Dynamin 2 Function and Animal Models for ADCNM

#### 3.2.1. Dynamin 2

Dynamin 2 (DNM2) is a large GTPase with membrane fission and tubulation activities. It is a key player during final steps of clathrin-mediated endocytosis to trigger the fission and release of vesicles. This mechanoenzyme is also implicated in membrane trafficking and recycling, cytoskeleton remodeling through interaction with actin and microtubules networks, apoptosis, and cytokinesis [83] (Figure 1).

In mammals, there are three genes encoding “classical” dynamins. While dynamin 2 is the only member ubiquitously expressed, dynamin 1 is mainly expressed in the brain, and dynamin 3 is particularly expressed in the brain, testis, and lung. Each dynamin has several splice isoforms. In the case of *DNM2*, exon 10a/b is mutually exclusive, while exons 12b and 13b are alternatively spliced [84,85]. Exon 12b was recently observed in mice and human tissues with predominant expression in skeletal muscle, which increased during muscle development [84]. In adult skeletal muscle, both *Dnm2* isoforms including exon 12b as well as the ubiquitous *Dnm2* isoforms (without exon 12b) are expressed.

Dynamin 2 is comprised of several functional domains (Figure 2) [83]. The N-terminal GTPase domain mediates the hydrolysis of GTP to GDP. GTP binding favors dynamin oligomerization, while GTP hydrolysis triggers dynamin oligomer disassembly [86]. The middle domain or stalk allows dynamin 2 dimerization in a cross-like fashion and is thus essential for its oligomerization. It may also bind to actin [87]. The Pleckstrin-homology (PH) domain binds to phosphoinositides such as PI(4,5)P_2_ and its affinity increases with dynamin polymerization. Dynamin 2 may also bind other PIs such as PI(3,4)P_2_, PI(3,4,5)P_3,_ and PI(3)P [88]. The GTPase effector domain (GED) (together with the middle domain) is involved in oligomerization and in regulating the GTPase activity through its interaction with the GTPase domain. The C-terminal proline-rich domain (PRD) mediates interaction with other proteins containing Src-homology domain 3 (SH3) such as endophilins, amphiphysins, sorting nexins, and syndapins. These interactions may regulate dynamin recruitment to the membrane or activity.

Dynamins oligomerize around tubular membrane templates, forming spiral structures before triggering membrane fission. Briefly, dynamin dimerizes through its middle/stalk domain, then dimers oligomerize to form a tetramer structure. This tetramer can have two conformations: open-active conformation or close-autoinhibited conformation. When the PH domain is flipped back on the middle/stalk domain, it forms an autoinhibitory state that is released upon binding to the lipid membrane by the PH domain [89]. Then, dynamin oligomerizes around membrane tubes, forming helicoidal structures, triggering membrane constriction upon GTP binding, and ultimately fission concomitant to GTP hydrolysis [83,90]. To date, several models have been proposed for the detailed mechanism of the action of dynamins [90].

DNM2 mutations have been associated with tissue-specific diseases and mainly concentrate in the middle/stalk and PH domains. A gain-of-function mechanism has been proposed for ADCNM mutations with a muscle-specific phenotype. Most ADCNM mutations linked to severe forms such as the common S619L missense are concentrated in the auto-inhibitory PH–stalk interface, while the common R465W mutation leading to a mild adult form is located in the tetramerization interface [89]. It is hypothesized that mutations in the PH–stalk interface open dynamin conformation and favors its oligomerization around membranes. In vitro studies have shown that ADCNM-mutants are hyperactive and form more stable oligomers [77,79,91]. More specifically, the S619L mutation behaves like a lipid unsensitized mutant, resulting in high GTPase activity independent of lipid binding [78,92].

#### 3.2.2. Mouse Models for DNM2-Related ADCNM

The first in vivo model for ADCNM was generated by homologous recombination and expresses the most frequent *DNM2* mutation observed in patients (*Dnm2*^R465W/+^) [93] (Table 3). This model mimics adult-onset cases with a slowly progressive myopathy as in patients. *Dnm2*^R465W/+^ mice have a normal lifespan and body weight. Impairment of contractile properties manifest from three weeks of age, while muscle atrophy progresses with age starting from two months. In muscle, a clear disorganization and central accumulation of mitochondria and reticulum is evidenced by oxidative staining from two months of age. Of note, centralized nuclei, sarcoplasmic strands, or type 1 fiber predominance characteristics of the human pathology were not seen. Mice homozygous for the mutation generally die on the first day of life; associated with mislocalized nuclei observed in 20% of fibers. Recently, a second ADCNM mouse model was described and reproduces the severe neonatal cases caused by the S619L mutation (*Dnm2*^S619L/+^) [94]. Some heterozygous mice die between E18.5 and postnatal day 10. Survivors present a normal lifespan with decreased body weight perhaps due to feeding difficulties. Impairment in motor function is observed from three weeks, and at eight weeks, histology shows fiber hypotrophy, abnormal central accumulation of oxidative activity, and a very slight increase in centralized nuclei (around 5% vs. 2% in WT). The main ultrastructural defect observed is the presence of enlarged and rounded mitochondria devoid of most cristae, potentially underlying the impaired muscle performance. This mitochondria anomaly was also confirmed in a patient with the same mutation. Similar to the *Dnm2*^R465W/+^ model, homozygous *Dnm2*^S619L/S619L^ mice do not survive past the first postnatal day. No defect in nerve conduction or structure was observed in both *Dnm2* knock-in models, although more specific electrophysiological studies will be required to totally exclude possible nerve involvement in these models.

Importantly, *Dnm2* homozygous knock-out is embryonically lethal [83], while specific knock-out in skeletal muscle leads to structural and metabolic defects of muscle unlike the CNM phenotypes [95]. Conversely, heterozygous *Dnm2*^+/−^ mice have a normal lifespan and do not display obvious phenotypes [96].

In addition to knock-in mouse models, transient exogenous overexpression of human *DNM2* CNM mutations in wild type (WT) mouse muscle with adeno-associated virus (AAV) was performed and correlated with a reduction in muscle force and CNM-like histological defects including fiber hypotrophy, nuclei mislocalization, and altered mitochondrial staining [97,98]. In detail, sarcomere structures were altered, neuromuscular junction (NMJ) was disrupted, and triads were abnormal with rounder and longitudinally oriented T-tubules and swollen sarcoplasmic reticulum. The structural defects found correlate with clinical severity observed in patients and knock-in mouse models with increased severity with the S619L mutation compared to the R465W mutation [98]. Interestingly, overexpression of WT DNM2 in muscle, either through AAV [97,98] or transgenesis [99], resulted in a clear increase in myofibers with centralized nuclei, central accumulations of oxidative staining, and decreased muscle performance, recapitulating the CNM phenotypes, and thus suggesting DNM2-related ADCNM mutations are gain-of-function in vivo [99].

#### 3.2.3. Other Animal Models for DNM2-Related ADCNM

The role of dynamin in endocytosis was first suggested through the study of the single dynamin ortholog, shibire gene drosophila mutants, presenting paralysis and depletion of vesicles in synaptic terminals at non-permissive temperatures [100,101]. Transgenic drosophila expressing human *DNM2* CNM mutants in muscle were also studied [77]. Similarly to mice, overexpression of WT and CNM mutants led to impaired locomotion, fiber hypotrophy, and fragmentation of T-tubules (Table 3).

Zebrafish: In developing zebrafish larvae, DNM2 exogenous expression of human *DNM2*-S619L induces defective motor function as seen by weaker swimming patterns [39,102]. Muscle structure displays abnormally located nuclei, perinuclear disorganization, and abnormal triads. Stimulus-associated intracellular calcium release is impaired and NMJs are disorganized. These findings were confirmed with transgenic lines overexpressing human DNM2 fused to enhanced GFP (EGFP), *Tg(DNM2-EGFP)* for WT-DNM2, and CNM-mutants (R465W, S619L) [103]. Similar to overexpression in mice, WT and CNM mutants provoked major muscle defects and impairment of motor function. Bragato et al. overexpressed the human DNM2-R522H CNM mutant in zebrafish and noted locomotion was severely impaired, muscles presented with increased centralized nuclei and decreased fiber size, and NMJ were altered [104].

Dog: The most common R465W DNM2 mutation was recently identified in a Border collie (*DNM2*^R465W/+^) with mildly progressive weakness and CNM histological hallmarks as myofiber hypotrophy, central nuclei, necklace fibers, and abnormal mitochondrial positioning (Table 3). Of note, histological anomalies preceded the clinical signs [105].

## 4. BIN1 and Autosomal CNM Forms

### 4.1. Disease Presentation and Genetics

BIN1-related centronuclear myopathy (BIN1-related CNM, OMIM#255200) is caused by mutations in *BIN1* encoding amphiphysin 2, a ubiquitous protein implicated in membrane curvature and tubulation (Table 1) [6]. Autosomal recessive CNM with childhood onset is caused by *BIN1* mutations that are probably loss-of-function [6,40,115,116,117] Autosomal dominant CNM cases caused by heterozygous *BIN1* mutation have also been reported with adult-onset and mild CNM [5]. The prevalence of BIN1-related CNM cases is estimated to be around 4% of total CNM patients and its worldwide incidence predicted to be seven newborns per year [9]. However, the finding of a specific *BIN1* mutation with a founder effect in Spanish Roma suggests that the incidence may be much higher in specific populations [116].

BIN1 patients present with a wide range of clinical presentations but are generally associated with a disease severity intermediate between the XLMTM severe neonatal form and the autosomal dominant adult-onset DNM2-ADCNM cases. Classical ARCNM cases have neonatal-childhood onset with more severe symptoms than ADCNM cases linked to BIN1 mutations, which present mild severity and adult onset. These are usually slowly progressive, and the prognosis is relatively good, even for neonatal cases in comparison with XLMTM. An exception is a few ARCNM cases with a splice mutation affecting the muscle-specific exon 11 of *BIN1* and with childhood onset and highly progressive myopathy [40].

BIN1-related ARCNM patients present muscle atrophy with diffuse muscle weakness together with facial weakness, ptosis, and ophthalmoplegia. Delayed motor milestones during childhood are common antecedents. Patients generally do not need ventilatory assistance. BIN1-related ADCNM cases present mildly progressive muscle weakness without facial weakness [5]. Other non-muscle phenotypes found in some patients are cardiac arrhythmia [115].

### 4.2. Amphiphysin 2 Function and Animal Models for BIN1-Related ARCNM

#### 4.2.1. Amphiphysin 2

Amphiphysin 2, also known as Bridging Interactor 1 (BIN1) or SH3P9, is a ubiquitous protein belonging to the BAR protein family of proteins that are involved in membrane trafficking and tubulation. It is implicated in several cellular functions including endocytosis, endosome recycling, cytoskeleton regulation, cell cycle progression, apoptosis, and DNA repair [118] (Figure 1).

In mammals, there are two different genes encoding for amphiphysins: amphiphysin1 (*AMPH1*), which is highly expressed in the brain, and amphiphysin 2 (*BIN1*), which is highly expressed in skeletal muscle (based on human GTEx data). *BIN1* has 20 exons, some of them alternatively splice to produce at least 12 tissue-specific isoforms with diverse functions [118]. Isoforms 1–7 are mainly expressed in the brain, the muscle-specific isoform 8 contains a polybasic domain, and other isoforms are rather ubiquitously expressed.

BIN1 has the following domains that present differently depending on the isoform (Figure 2) [118]. The Bin1/Amphiphysin/Rsv (N-BAR) domain is present in all isoforms and is encoded by exon 1 to 10, although exon 7 is not always included. Its homo- or hetero-dimerization stabilizes BIN1 oligomerization, forming a positive concave face that binds to negatively charged lipids, and thus conveys membrane curvature sensing. The N-terminus amphipathic alpha helix inserts into the membrane and contributes to curvature generation [119,120]. The phosphoinositide (PI) binding domain is encoded by the muscle specific exon 11 (initially named exon 10 from cDNAs as it is never found with exon 7) [33,121] and corresponds to a short polybasic sequence that increases BIN1 affinity toward negatively charged lipids such as PI(4,5)P_2_, PI(3)P, or PI(5)P [122,123]. It was shown to enhance membrane tubulation in cells [6,123] and also regulate BIN1 conformation by binding to the SH3 domain into a “folded” conformation, potentially preventing further access to the SH3 domain by interactors [124]. The clathrin and AP2 binding (CLAP) domain is encoded by exons 13–16, which is important for endocytosis and is specifically present in the brain but not in muscle isoforms [125,126]. The Myc binding domain (MBD) is encoded by exons 17–18 [127]. Exon 17 is not always present in adult skeletal muscle isoforms [33,121] The Src homology 3 (SH3) domain is present in all isoforms and encoded by the last exons 19–20. It is implicated in protein–protein interactions by binding to their proline rich domain (PRD) [128]. Protein binding partners include dynamin, synaptojanin, N-WASP, and Nesprin.

CNM homozygous missense mutations in the BAR domain were shown to impair its membrane tubulation properties. D151N and R154Q mutations are predicted to decrease the ability to form a dimer and bind phosphoinositides [6,117]. The K35N mutation decreases the number of positively charged amino acids of the N-terminal amphipathic helix, decreasing the ability of BIN1 to sense and generate membrane curvature [6]. Moreover, a splice site mutation skipping the muscle specific in-frame exon 11 removes the PI binding domain and decreases membrane tubulation [40]. This mis-splicing was also observed in patients with myotonic dystrophy 1 (DM1) due to alterations in splicing in the context of that disease [122]. Other ARCNM mutations truncate the SH3 domain (as K573X or K575X) and affect binding to DNM2, MTM1, and N-WASP, but do not impair membrane tubulation [6,129,130].

The heterozygous mutations causing ADCNM are different and encompass two mutations in the N-terminal amphipathic helix (K21del and R24C) and three stop-loss mutations causing the addition of 52 amino acids following the SH3 domain [5]. Of note, *Bin1* heterozygous knock-out mice present no phenotype [131] and heterozygous carriers of *BIN1* recessive mutations are not affected [5], suggesting dominant and recessive *BIN1* mutations have a different functional impact. Further in cellulo studies were undertaken to understand the difference between dominant and recessive mutations in the N-BAR domain. Expression of BIN1 mutations in COS cells impaired the formation of BIN1-induced membrane tubules. However, co-expression of WT-BIN1 allowed for the recruitment of recessive mutants to the membrane tubules, but dominant mutants were not recruited. It was then hypothesized that recessive mutations induce a partial loss-of-function whereas dominant mutations lead to a full loss-of-function or a dominant-negative effect.

#### 4.2.2. Mouse Models for BIN1-Related ARCNM

No animal models carrying the patients’ mutations have been described up to now. However, several animal models with the deletion of an in-frame exon or full knock-out of *Bin1* have been generated. The first constitutive *Bin1* KO (*Bin1*^−/−^) was described in 2003, with partial deletion of exon 3 and no exons 4 and 5 [132] (Table 4). This led to perinatal death during the first day of life. The cause of death was not completely clear, but defects in heart with aberrant sarcomere structure and ventricular cardiomyopathy were proposed. However, cardiomyocyte-specific loss of *Bin1* causes a dilated cardiomyopathy beginning by 8–10 months of age [133]. More recently, a mouse model lacking the last exon 20 (*Bin1e*x20^−/−^), causing truncation of the SH3 domain similarly to some CNM patients, has been described [84,131]. This deletion also leads to perinatal death, although no cardiac defects have been found. The pups probably die due to feeding problems as strong hypoglycemia was noted at day 1 [131]. Skeletal muscle-specific deletion of the same exon induced by a Cre recombinase under the control of the HSA promotor (*Bin1e*x20^hsa−/−^) also causes perinatal death. Altogether, these data suggest BIN1 is necessary for perinatal maturation of skeletal muscle, but dispensable for cardiac muscle development [131]. The study of the skeletal muscle of *Bin1e*x20^−/−^ newborns showed centralized nuclei, central collapse of oxidative activity, and altered triad structures, reminiscent of CNM histopathology. Inducible deletion of BIN1 in adult skeletal muscle (*Bin1e*x20^hsa(i)−/−^) with 80% reduction in BIN1 protein level does not cause histological defects, or abnormal muscle ultrastructure or triad structures, suggesting that BIN1 is mainly necessary for muscle maturation [131]. However, acute downregulation of *Bin1* in the flexor digitorum brevis (FDB) muscle with shRNA promotes disruption of the T-tubule structure and alterations in intracellular Ca^2^⁺ release [134].

As skipping of the muscle-specific exon 11 of *BIN1* causes ARCNM with highly progressive myopathy [40], a mouse model with deletion of *Bin1* in-frame exon 11 (*Bin1e*x11^−/−^) was generated, causing an isoform switching from the muscle-specific isoform 8 (with exon 11) to the ubiquitous isoform 9 (without exon 11) in skeletal muscle [131]. This switch does not alter normal muscle structure and function, but impaired muscle regeneration induced by injury. A slight decrease in T-tubule network was quantified with no impact on the excitation–contraction coupling (ECC). However, a similar *Bin1* mis-splicing was reproduced in adult muscle by injection of U7-exon 11 antisense and this was sufficient to induce T-tubule alterations with an impact in muscle force [122]. Altogether, these mouse models lead to better understanding of the BIN1 function in muscle, pointing to BIN1 as a key player for muscle maturation, and is required for muscle function and regeneration in adulthood.

The disadvantages of these previously described models were the early lethality or absence of a clear muscle phenotype, preventing their use for testing therapies. Recently, a viable mouse model muscle-specific knock-out of *Bin1* was described and faithfully reproduces CNM motor and histological phenotypes [135]. The ubiquitous exon 20 was deleted from 17 days post-coitum (dpc) in muscles with a Cre recombinase under the control of the muscle creatine kinase (MCK) promotor (*Bin1e*x20^mck−/−^) [135]. In comparison, thasHSA promoter used to obtain perinatal lethal mice expresses from 9 dpc. Mice presented a normal lifespan and developed a progressive myopathy with decreased muscle force at eight weeks of age. In muscle sections, histological CNM-hallmarks were observed as abnormal mitochondrial distribution with central accumulation of oxidative activity and fiber hypotrophy. *Bin1*ex20^mck−/−^ muscle fibers present defects in the T-tubule network and impaired ECC, which are probably the main contributors to the muscle weakness observed in this mouse model. All CNM hallmarks had worsened by four months of age. However, no centralized nuclei were observed until 12 months of age (maximum age analyzed).

#### 4.2.3. Other Animal Models for BIN1-Related ARCNM

Zebrafish: *bin1* morphant zebrafish also revealed common features observed in muscle biopsies from CNM patients [136] (Table 4). They presented with dorsal curvature and S-shape (reminiscent to kyphosis/scoliosis seen in CNM patients) and a deficit in early motor function. Histology showed mis-localized and grouped nuclei and disorganized myofibers. Unusual membranous whorls, as observed in the dogs, were also observed by electron microscopy as well as abnormal triads. Interestingly, overexpression of BIN1 with or without exon 11 rescue abnormal triads, again supporting that exon 11 is not essential for muscle development.

Dogs: A spontaneous canine model with exon 11 mis-splicing was described in 2013 [40] (Table 4). A group of Great Danes presented a rapidly progressive myopathy named as Inherited Myopathy of Great Danes (IMGD), characterized by muscle atrophy and exercise intolerance with an onset at about six months of age [137,138,139]. They were affected by an homozygous *BIN1* mutation at the exon 11 AG acceptor splice site that leads to exonization of part of intron 10 and loss of BIN1 protein [40]. The dogs reproduced the histopathological hallmarks found in patients, with increased internalized nuclei, muscle atrophy, and membrane abnormalities such as aberrant triads, and accumulation of membranous structures.

## 5. RYR1 and Autosomal Recessive Centronuclear Myopathy

### 5.1. Disease Presentation and Genetics

RYR1-related autosomal recessive centronuclear myopathy (RYR1-related ARCNM, OMIM#255320) is caused by mutations in the *RYR1* gene encoding the ryanodine receptor, which is the main sarcoplasmic reticulum (SR) calcium release channel in skeletal muscle [8] (Table 1). Recessive mutations in *RYR1* cause different albeit overlapping myopathies including multi-minicore disease (MmD), congenital fiber type disproportion, or CNM. Dominant mutations in this gene have been associated with central core disease (CDD, OMIM#117000), King–Denborough syndrome, and malignant hyperthermia (MHS, OMIM#145600). In the present review, we focused on recessive mutations causing CNM-like phenotypes. Recessive cases are normally caused by compound heterozygous mutations in *RYR1*, with one mutation causing premature termination that may reduce RyR1 protein expression and a second heterozygous *RYR1* missense mutation, resulting in a dramatic decrease in the overall RYR1 protein levels. [7,8]. The prevalence of ARCNM cases associated with *RYR1* mutations represents about 12% of CNM patients. The incidence of global is estimated in 21 newborns per year [9].

RYR1-related ARCNM patients generally present at birth or with childhood onset. Severity is usually intermediate between the severe form of XLMTM and mild DNM2-related ADCNM cases. Patients are profoundly weak and hypotonic at birth, but most improve over time, making the prognosis better than for XLMTM. It is clinically characterized by proximal muscle weakness, hypotonia, reduced or absent deep tendon reflexes, associated with ophthalmoplegia with or without ptosis [22]. Some patients require ventilatory assistance and generally suffer frequent respiratory tract infections. Other comorbidities commonly found are thoracic deformities and spinal involvement. The prognosis of the disease is relatively good in comparison with XLMTM, even in neonatal cases, as they showed improvements over time.

In the first described cohort of patients with RYR1-related ARCNM, muscle histology was characterized by fibers with centralized and internalized nuclei, but to a lower extent than other genetic forms of CNM [8]. Muscle biopsies presented predominance of hypotrophic type 1 fibers with hypertrophy of type 2 fibers. Oxidative staining showed abnormalities with central accumulations and some cases with peripheral halos depleted of oxidative activity resembling XLMTM histology. Most patients developed cores on follow-up biopsies [7]. In another cohort of seven unrelated patients with heterozygous compound mutations, muscle biopsies showed a significant number of fibers with internalized nuclei and core-like structures [7]. However, they were distinguishable from classical “cores”, because these areas with depleted oxidative staining were larger, more diffuse, and had no limited boundaries. Ultrastructural studies commonly reveal Z-disk streaming in those large areas with myofibrillar disorganization and accumulation of Z-disk proteins [7]. These main RYR1 disease features were confirmed more recently in other cohorts [42]. Overall, the clinic and histopathology of RYR1-related ARCNM mostly represent a continuum between classical CNM and core myopathies, with core-like structures and internal rather than central nuclei. Its diagnosis requires a muscle biopsy, apart from the genetic confirmation.

### 5.2. RyR1 Function and Animal Models for RYR1-Related ARCNM

#### 5.2.1. Ryanodine Receptor 1

Ryanodine receptor 1 (RyR1) is a large transmembrane Ca^2+^ release channel, located at the terminal cisternae of the sarcoplasmic reticulum in close apposition with the T-tubules, in a specific structure of the muscle called the triad. It mediates excitation–contraction coupling (ECC) by releasing calcium from the SR into the cytosol in response to muscle fiber stimulation by the motor neuron at the neuromuscular junction (NMJ). RyR1 was described to mediate several cell functions as a regulator of calcium concentrations and redox homeostasis, and gene expression as an epigenetic modulator [140] (Figure 1).

In mammals, there are three different ryanodine receptors encoded by three different genes, with tissue-specific expression: RyR1 is predominantly expressed in skeletal muscle, while RyR2 encodes the cardiac ryanodine receptor and RyR3, originally identified in the brain, is expressed in many tissues including skeletal muscle at relatively low levels. RyRs are homotetramers with a total molecular weight higher than 2.2 MDa. Briefly, it consists of an ion conductive pore formed by its C-terminal transmembrane domain and is regulated by its large N-terminal cytoplasmic domain (for more details see [141,142], Figure 2). The cytoplasmic domain of RyR1 can interact with Ca^2+^ and Mg^2+^ ions, ligands such as caffeine, ATP, and ryanodine, and accessory proteins as calmodulin and STAC3 [142]. RyR1 also directly interacts with the voltage-gated Ca^2+^ channel (Ca_v_1.1; DHPR) located at the T-tubule. In the SR lumen, it indirectly connects with the Ca^2+^ buffering protein calsequestrin. Most RyR1 mutations identified to date are located clustering in 3–4 different large hotspots along the gene, although new mutations are also increasingly being identified outside of those regions [142].

#### 5.2.2. Mouse Models for RYR1-Related ARCNM

Both dominant (or de novo) and recessive mutations have been reported in RYR1-related myopathies. We focused here on models generated to reproduce recessive mutations, as in patients with ARCNM (Table 5). The two main pathophysiological mechanisms of RyR1 diseases are either alteration of calcium channel permeability or reduction in the amount of protein. A comprehensive review of the different preclinical models available for RYR1-related myopathies was recently published [143]. Models described below for compound heterozygous *RYR1* mutations carry one frameshift mutation together with a missense mutation in the other allele, resulting in low levels of RyR1 proteins with mono-allelic expression of the missense mutation.

A mouse model mimicking recessive cases of RYR1-related myopathy with early clinical onset was generated using the CRISPR/Cas9 gene-editing strategy [144]. One allele contains the patient point mutation T4706M and the second allele corresponds to a 16 base pair frame-shift deletion, resulting in a premature stop codon (*Ryr1*^TM/Indel^). *Ryr1*^TM/+^ and *Ryr1*^Indel/+^ mice were phenotypically indistinguishable from WT mice, and the combination of both alleles resulted in a high reduction in RyR1 protein level (80%). *Ryr1*^TM/Indel^ mice present a motor phenotype from 14 days of age, with a decrease in body weight and a median survival of 42 days. At the end of their life, they exhibit reduced movement, muscle weakness, hindlimb paralysis, and severe scoliosis. The probable cause of death is respiratory failure caused by the combination of muscle weakness and spine curvature changes (commonly observed in patients). Muscle biopsies exhibited hypotrophy in type I and type II fibers without changes in fiber type and no evidence of cores in oxidative activity. There was a modest albeit significant increase in centralized nuclei (0.8%). Specific muscle force was reduced probably due to ECC defects as mice exhibited reduced twitch and tetanic calcium release.

In parallel, another mouse model carrying compound heterozygous recessive *Ryr1* mutations was generated by homologous recombination [145] (Table 5). It harbors a frameshift mutation (Q1970fsX16 in exon 36) together with the missense mutation A4329D (in exon 91) and is named *Ryr1* ^Q1970fsX16/A4329D^. It recapitulates the main features found in multi-minicore patients. Mice had decreased body weight and spontaneous movement from 20 weeks of age. Specifically, muscle force was decreased, together with a reduction in electrically evoked Ca^2+^ transients. Skeletal muscle biopsies showed the presence of cores with myofibrillar disorganization and misplacement of mitochondria. The combination of these two mutations resulted in a reduced RyR1 protein level by around 65%.

As a common feature of diseases caused by recessive *RYR1* mutations is a pronounced decrease in the RyR1 protein, several models were generated to shed light on the pathophysiological effect of the RyR1 protein level reduction. The full deletion of *Ryr1* expression in mice (skr^m1^/skr^m1^) induces perinatal lethality, possibly because of respiratory failure due to the lack of ECC and muscle contraction [146]. A severe decline in muscle mass as well as skeletal muscle abnormalities including abnormal junctions between SR to T-tubules was observed [147]. Recently, inducible skeletal muscle specific *Ryr1* deletion (*Ryr1-Rec)* at the adult stage was characterized [43]. Protein level was reduced to maximum 50% of the initial level, with more pronounced reduction in mRNA (80%). This correlates with progressive muscle weakness and atrophy together with defective ECC and reduced calcium fluxes. This mouse model recapitulates main features observed in a group of patients with central core disease and similar RyR1 protein reduction [148]. Main observed features were muscle atrophy, abnormal mitochondrial distribution, fiber remodeling, inhibition of autophagy, and increased protein expression of proteins implicated in calcium handling and muscle structure including STIM1 and desmin. Of note, the *Ryr1*^+/−^ mice did not present muscle weakness or reduced Ca^2+^ release, although only a 15% decrease in protein was achieved [149]. Overall, it seems that the reduction in RyR1 protein to at least 50% is necessary to observe functional defects in mice.

#### 5.2.3. Other Models for RYR1-Related ARCNM

Zebrafish: A recessive zebrafish model of RYR1-related myopathy named *relatively relaxed* (*ryr^mi340^*) was characterized in 2007 [150] with a mutation in *ryr1b* (expressed in slow and fast twitch muscles) (Table 5). The mutation (32-bp insertion) led to decreased mRNA of *ryr1b*, resulting in a slow swimming phenotype due to weak muscle contractions. The defect in contraction can be explained by defective ECC as Ca^2+^ transients induced by depolarization are decreased in fast muscles. The absence of RyR1 leads to defects in DHPR localization at T-tubule–SR junctions. Ultrastructural studies revealed small cores and swollen sarcoplasmic reticulum in fast muscle. Increased oxidative stress was also observed [151]. A *ryr1a*;*ryr1b* double-mutant zebrafish model have recently been generated, resulting in complete paralysis of embryos and early death [152].

## 6. Common Pathomechanisms of CNMs

**Figure 3:**
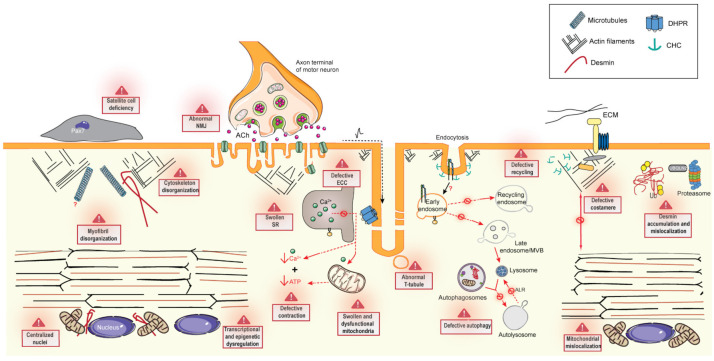
Schematic illustration of pathogenic alterations in skeletal muscle caused by mutations in MTM1, BIN1, DNM2, and RYR1. ECM (extracellular matrix), ALR (autophagic lysosome reformation), SR (sarcoplasmic reticulum), Ub (ubiquitin), NMJ (neuromuscular junction), ECC (excitation-contraction coupling), Ach (acetylcholine), MVB (multivesicular bodies). The figure uses modified images from Servier Medical Art Commons Attribution 3.0 Unported License (http://smart.servier.com, accessed on 29 August 2021).

In the past years, in-depth studies of patients and cellular and animal models have led to a better understanding of the mechanisms involved in the different CNM forms, and highlighted common altered pathways (Figure 3). Moreover, transcriptomic studies of CNM mouse and dog models were able to underline a common disease signature and potential biomarkers [153,154]. A better understanding of the pathomechanisms is useful to identify therapeutic targets.

### 6.1. Excitation–Contraction Coupling and the Triad

Skeletal muscle contraction is triggered by excitation–contraction coupling (ECC) that occurs at the triad and is leading to increased cytosolic Ca^2+^ concentration. The triad is composed of the juxtaposition of a transversal invagination of the plasma membrane called a T-tubule and two sarcoplasmic reticulum (SR) cisternae [58]. ECC relies on the tight control of SR Ca^2+^ release via RyR1, which is directly activated by DHPR upon T-tubule depolarization. Defects in ECC and calcium homeostasis are a common feature of CNM and probably the main cause for the observed muscle weakness.

One of the first models showing amphiphysin is essential for the maturation of T-tubules is the amphiphysin null mutant in drosophila (*amph*^mut^), ortholog for amphiphysin-1 (AMPH1) and amphiphysin-2 (BIN1) in mammals [155]. Flies were fertile and viable but flightless with T-tubule malformations. Triad alterations were also observed following a decrease in BIN1 in muscle in a spontaneous CNM dog model [40]. Interestingly, skipping of the skeletal muscle-specific exon 11 in patients promoted a progressive and severe CNM while this isoform switch from the muscle to the ubiquitous isoform in muscle in the *Bin1*ex11^−/−^ mouse correlated with a slight decrease in the T-tubule network without a detectable impact on ECC [40,131]. Defects in T-tubule formation were described in mice lacking BIN1, and *Bin1*ex20^−/−^ primary myotubes displayed no T-tubules connected to the plasma membrane [131]. Impairment of BIN1 in skeletal muscle, following deletion of exon 20, triggered during early embryogenesis at E9 (HSA-Cre; *Bin1*ex20^hsa−/−^) or later from E17 (MCK-Cre; *Bin1*ex20^mck−/−^) led to either perinatal lethality or viability, respectively [131,135]. This difference supports that BIN1 is important for T-tubule formation as T-tubules start to form from E15 [156]. Muscle fibers from *Bin1*ex20^mck−/−^ mice presented T-tubules, but at decreased density and impaired DHPR current and SR Ca^2+^ release (60% decrease), suggesting that BIN1 is also important for the late maturation of T-tubules [135]. Acute downregulation of *Bin1* with shRNA in adult mice led to a defective T-tubule network linked to alterations in intracellular Ca^2+^ release [134], while no impact in triad structure was noted after the deletion of BIN1 by tamoxifen induction (*Bin1e*x20^hsa(i)−/−^) [131]. Similarly, skipping of *Bin1* exon 11 using a U7 small nuclear RNA significantly reduced muscle force, correlating with extensive T-tubule disorganization [122].

*Mtm1* deficiency in mice leads to an abnormal triad shape, T-tubule orientation, and a reduced number of triads per sarcomere. Abnormal localization and expression of DHPR and RyR1 were also observed [67]. Abnormal triad structure and reduced DHPR and RyR1 expression are features also found in myotubular myopathy patients [29,34,157]. At the functional level, ECC was impaired with a reduced peak amplitude of voltage-activated SR Ca^2+^ release and delayed activation kinetics [67,70,71]. Abnormal triad and defective ECC was also found in zebrafish myotubularin morphants [34]. In addition, dilated SR at the triad was noted in *Mtm1*^−/y^ mice. Subcellular fractionation suggests MTM1 is mainly associated with SR membranes at the triad and that its enzymatic activity is necessary for SR remodeling [158]. PI(3)P was proposed to be important for promoting SR membrane curvature in skeletal muscle [158].

Similar investigations were conducted in the *Dnm2*^R465W/+^ mouse model, where muscle weakness appeared before fiber hypotrophy and histological abnormalities were present [93]. Altered calcium homeostasis (abnormal cytosolic Ca^2+^ concentration) was observed with muscle weakness [93,110], although the T-tubule network appeared unaltered unlike in *Mtm1*^−/y^ mice. Muscle fibers exhibited alteration in the DHPR Ca^2+^ current and altered Ca^2+^ release by RyR1 to a lower extent than the *Mtm1*^−/y^ fibers (30% vs. 60% decrease), correlating with the lower force of *Mtm1*^−/y^ muscle. Spatial non-uniformity of Ca^2+^ release and spontaneous Ca^2+^ sparks were observed in both *Dnm2*^R465W/+^ [112] and *Mtm1*^−/y^ fibers [70,71]. Spontaneous Ca^2+^ sparks are uncommon events in skeletal muscle, which occur when the RyR1 channel is opened by Ca^2+^ independently of the DHPR control (Ca^2+−^ induce Ca^2+^ release). The DNM2 mouse model (*Dnm2*^S619L/+^) with a more severe reduction in muscle force showed only a slight triad misalignment [94]. Interestingly, overexpression of human *DNM2* (WT or CNM mutants) in mice, drosophila, or zebrafish led to a decreased number of triads per sarcomere and swollen and longitudinally oriented T-tubules [77,97,98,102].

RyR1 heterozygous compound mutations in mice led to reduced RyR1 protein levels and muscle contraction defects due to defective ECC, resulting in reduced SR Ca^2+^ release. For example, the *Ryr1*^TM/Indel^ mouse presented a pronounced decrease in RYR1 protein level (80%) and decreased DHPR protein level. Detailed studies showed abnormal intracellular calcium dynamics, with significant reduction in twitch and tetanic evoked Ca^2+^ release, resulting in reduced specific force production [144]. Similarly, the recessive *Ryr1*^Q1970fsX16/A4329D^ mouse model showed reduced electrically evoked Ca^2+^ currents, although no changes in DHPR protein expression were found [145]. In *Ryr1-Rec* mouse (muscle-specific *Ryr1* deletion), the current via DHPR and the calcium influx via RyR1 were both reduced, while no changes in DHPR expression or alterations in the T-tubule network and density were noted [43]. This apparent discrepancy may be explained by a “retrograde coupling” where RYR1 controls DHPR function [159]. RyR1 has no membrane remodeling action per se, but may control the triad structure and the correct positioning and localization of DHPR at the T-tubule. Its decrease leads to defects in DHPR expression and localization at the T-tubule [144,150] and total absence causes an abnormal junction between the SR and T-tubule [147]. The expression level of other proteins implicated in calcium handling may also be altered. For example, ORA1, STIM1, and CLIMP63 expression is increased after RYR1 depletion; as these proteins are known to be implicated in reticulum morphogenesis, it may explain the formation of abnormal multiple triads in RyR1 mouse models and in patients [148].

It is important to highlight that as Ca^2+^ is a second messenger, aberrant calcium homeostasis due to impaired ECC can also induce epigenetic and transcriptional changes as well as modulate intracellular pathways. Zebrafish *ryr* mutants with reduced expression of several *ryr* transcripts (*ryr1a*, *ryr1b* and *ryr3*) and complete loss of protein showed altered intracellular calcium that impacts on tissue patterning in embryos by blocking Shh signaling essential for normal development [160]. Epigenetic dysregulation with increased expression of histone deacetylases was previously found in patients and mouse models with RyR1 mutations [44,145]. Upregulation of histone deacetylase-4 was also observed in muscle biopsies from XLMTM patients [29].

While RyR1 is the main calcium channel of the SR, MTM1, DNM2, and BIN1 have been suspected to play a key role in T-tubule formation and maintenance. In muscle biopsies, MTM1 is enriched at the triads on both sides of the Z-line and associated with SR membranes [158]. BIN1 locates to or in the vicinity of T-tubules [118,125]. DNM2 is colocalizing with BIN1 longitudinally in immature muscle, but is mainly found at the Z-line in close proximity to the triad in mature muscle [84,97]. Mutations in these three proteins were shown to induce abnormal localization of BIN1, DHPR, and RYR1 in patients [22,33]. MTM1 may recruit BIN1 and DNM2 to specific muscle membranes. MTM1 can directly bind BIN1 and BIN1 binds DNM2. In cells, MTM1 directly binds BIN1 and enhances BIN1 membrane tubulation properties [130], while BIN1 appears to bind and negatively regulate DNM2 in vitro and probably in vivo [84,161]. Moreover, MTM1 and DNM2 can be recruited to membrane tubules induced by BIN1 overexpression, while DNM2-CNM mutations promote tubule fragmentation [6,77,102,123,161]. There is some evidence in cardiomyocytes and neurons that BIN1 and dynamin regulates CaV1.2 (DHPR) transport to the plasma membrane or T-tubule [162,163]. Finally, RyR1 was described to bind to phosphoinositides [164], while modulation of the phosphoinositide level with PI3K blockers such as wortmannin rescued the Ca^2+^ release defect of *Mtm1*^−/y^ fibers [70]. Overall, it is possible that the phosphoinositide substrates and product of MTM1 control both membrane remodeling and the activity of RyR1 at the triad.

### 6.2. Cytoskeleton Regulation and Organelle Positioning

#### 6.2.1. Cytoskeleton Regulation

A highly organized intracellular network of membrane organelles and cytoskeletal systems are important for a proper sarcomere structure and muscle contraction. There are three main cytoskeletal components regulating spatial organization in the cell and regulating the position and movement of organelles in a dynamic way: microfilaments (MFs) or actin filaments, microtubules (MTs), and intermediate filaments (IFs).

Actin. Actin cytoskeleton is a key player for endocytosis, nuclei positioning, NMJ formation, costamere maintenance, and triad organization. MTM1, DNM2, and BIN1 have been reported to have direct or indirect impact on actin remodeling.

MTM1 can be recruited to plasma membrane “ruffles” or actin rich membrane protrusions induced by the activation of the Rho-GTPase RAC1 in HeLa cells. This was shown to be dependent on its RID domain [53]. RAC1 is a small GTPase involved in the regulation of actin dynamics, and is responsible for the activation of the regulator of actin polymerization, N-WASP [165]. In addition, the *C. elegans* ortholog *mtm-1* was described to interact with the guanine nucleotide exchange factor (GEF) complex regulating RAC1 to control cytoskeleton rearrangements during apoptotic cell clearance [166]. Further implications of MTM1 in actin dynamics in muscle have not been described in detail to date. DNM2 directly binds actin filaments (F-actin) and regulates the actin cytoskeleton dynamics by interaction with proteins implicated in actin polymerization [167,168] or by bundling and organizing actin filaments [87]. Indeed, myofibers from *Dnm2*^R465W/+^ presented altered actin organization and decreased polymerization [111]. Correct actin remodeling is important for endocytosis, NMJ development, but also essential for costamere organization. Costameres are the structural-functional component of striated muscle connecting the sarcomeres to the cell membrane and to the extracellular matrix through integrins [169,170]. Overall, costameres maintain sarcomere structure and adhesion to allow an efficient transmission of muscle force. It is composed of complexes of transmembrane proteins including the focal adhesion complex and dystrophin–glycoprotein complex together with the cytosolic proteins vinculin, FAK, or talin [170]. Vassilopoulos et al. characterized a new complex formed by clathrin plaques, anchoring desmin filaments, and regulated by the clathrin adaptor AP2 together with DNM2 and the actin nucleating-promoting factor N-WASP [109,171]. DNM2 was required to organize desmin and actin around those clathrin plaques and its depletion in myotubes led to costamere disorganization and desmin aggregation [109]. DNM2 was previously implicated, together with clathrin, in the regulation of actin dynamics for focal adhesion turnover and maintenance [172,173]. Costamere dysfunction is also linked to defective recycling as later described in the membrane trafficking section.

BIN1 was shown to regulate actin by direct binding to F-actin through its BAR domain [174] and by recruiting actin-regulating proteins as N-WASP through its SH3 domain [129]. This function appears to be involved in triad organization and maintenance and nuclei positioning [41,129]. Of note, N-WASP localization is disrupted in muscle biopsies from patients with DNM2 and BIN1 mutations and to a lesser extent in patients with *MTM1* mutations [129]. More generally, BAR domain proteins have been shown to regulate cytoskeletal organization through the regulation of Rho GTPases [175].

Microtubules. Microtubules are implicated in the cell organization, intracellular transport, and nuclei positioning. In more detail, when myoblasts fused to form myotubes, nucleation of microtubules changes from the centrosome to the nuclear envelope. This event, together with other proteins, drives nuclear movements [176]. Dynamin was originally discovered in 1989 as a protein binding to microtubules [177]. It was then shown how microtubules activate dynamin GTPase activity, although the physiological impact of this regulation is still unknown [178]. DNM2 was suggested as a possible regulator of microtubule polymerization–depolymerization as DNM2 depletion in COS cells increased the level of the acetylated tubulin, stabilizing the formation of microtubules [179]. In COS-1 cells, overexpressed DNM2-CNM mutants showed decreased colocalization with microtubules, while no defects in microtubule dynamics and organization was observed in the *DNM2*-related ADCNM patients’ fibroblasts [180]. In addition, BIN1 was described to bind to CLIP170, a microtubule + end binding protein, to regulate nuclei positioning [41].

Intermediate filaments. Desmin is one of the major cytoplasmic intermediate filaments in the skeletal muscle and maintains mechanical integrity during contraction [181]. Mutations in desmin cause desmin-related myopathy (OMIM#601419) and cardiomyopathy (OMIM#604765). Desmin localizes to Z-disk, where it plays a key role in the integration and maintenance of the structure and function of striated muscle [181]. Specifically, desmin anchors several structures (mitochondria, nucleus, and Z-disk) to the sarcolemma cytoskeleton at the costamere and regulates peripheral nuclei positioning in myofibers [182]. Desmin localization and expression were found to be commonly dysregulated in patients with mutations in *DNM2* [22,109], *MTM1* [22,31,32], *BIN1* [22], and *RYR1* [43]. Desmin accumulated in the center of the myofibers.

MTM1 interacts with desmin specifically in skeletal muscle and regulates its assembly and proper localization [31]. *Mtm1* depletion in muscle leads to increased levels of desmin that form insoluble aggregates. Expression and localization of desmin were reestablished after AAV-mediated MTM1 expression. Interestingly, this was also rescued by the overexpression of phosphatase-dead myotubularin (MTM1-C375S), suggesting that desmin aggregation is independent of the phosphatase activity of MTM1 [183]. Desmin protein level was found increased in another *Mtm1* deficient mouse model and rescued by autophagy activation [61]. Desmin aggregation in *Mtm1*^−/y^ myofibers was also rescued by *Dnm2* downregulation [184]. Slight desmin alterations have been observed in *Dnm2*^R465W/+^ fibers and linked to defective costameres [109]. This abnormal desmin localization was also observed in muscle from *Bin1e*x20^mck−/−^ and also rescued after DNM2 reduction [135]. Finally, desmin protein increase and abnormal central accumulation were also found in muscle fibers from the *Ryr1*-Rec mouse line with adult deletion of *Ryr1* [43].

#### 6.2.2. Organelle Positioning

Nuclei position. Alteration of the cytoskeletal network correlates with organelle mispositioning. In particular, abnormal nuclei positioning is a key hallmark in CNM patients and does not correlate with excessive muscle regeneration, unlike that of dystrophies. Of note, nuclei misposition is not consistently reproduced in the different CNM mouse models.

During muscle development, following myoblast fusion, the nuclei initially moved to the center of the fiber (nuclear centralization), and then spread along its axis (nuclear spreading) to finally translocate to the periphery where it is distributed regularly along the sarcolemma (nuclear dispersion), defining a volume of cytoplasm controlled by gene transcription from a single nucleus called the myonuclear domain (MND). Following this step, some nuclei remained clustered under the NMJ (nuclear clustering) as described in detail in [176]. It was discovered that during this process, there is a switch from microtubule (MT)-driven to actin-driven nuclear positioning [176]. Interestingly, the role of BIN1 in nuclei positioning is conserved through evolution and in different tissues. Indeed, the *Caenorhabditis elegans amph-1* (BIN1 ortholog) null mutant (*amph-1*(tm1060)) displays nuclear mispositioning in intestine and seam cells [41]. In this organism, amphiphysin has been proposed to link the nuclear envelope through direct binding to Nesprin with both actin and microtubule cytoskeletons through actin and CLIP-170 [41]. BIN1 mutations in the SH3 domain disrupt N-WASP interaction, participating in nuclei mispositioning [129]. Patient biopsies from BIN1 and DNM2-CNM, and to a lesser extent, XLMTM, showed impaired organization of N-WASP [129]. In addition, desmin has been described as a key player in the positioning of non-synaptic nuclei acting as a repellent between nuclei, leading to their proper distribution along the periphery of the fiber [185,186]. Desmin aggregation commonly observed in CNM could impact proper nuclei positioning.

In situations where nuclei are mispositioned such as in CNM, myonuclear domains can be disrupted, causing muscle dysfunction. In *Dnm2*^R465W/+^ mouse myofibers, no major anomalies in myonuclear morphology and ultrastructure were described, while an impairment in the spatial distribution of nuclei was identified [108]. The number of myonuclei is decreased together with fiber size, contributing to the maintenance of the MNDs. On the other hand, in *Mtm1* deficient mice, dogs, and human muscles, a smaller size of MND was found, together with myonuclei misposition [75]. These phenotypes correlated to decreased myofilament force production, most probably contributing to muscle weakness. Conversely, regenerating muscles with a high number of centralized nuclei displayed normal contractile function. The authors proposed that the decrease in global nuclear synthetic activity led to reduced expression of contractile protein (e.g., actin and myosin), contributing to muscle weakness [75].

It is thus still unclear if centralization of nuclei in CNM muscles is causing disease. In XLMTM patients, it was described that the percentage of centralized nuclei was highly variable and did not correlate to disease severity [187]. In addition, nuclei internalization was not reproduced in several CNM mouse models displaying muscle weakness [93,94,135].

Mitochondrial position and function. Mitochondrial mispositioning is a feature shared by all CNM forms and has been observed in patients as well as in different animal models. Mitochondrial shape defects were also reported. Mitochondrial enlargement was described in murine muscles expressing the DNM2-R465W mutant [93,97,98]. In the severe *Dnm2*^S619L/+^ mouse model and patients with the same mutation, swollen mitochondria with disrupted cristae were noted in skeletal muscle, but not in liver or heart [94]. Similarly, enlarged mitochondria lacking cristae were observed in murine muscles overexpressing the DNM2-S619L mutant [98]. At the functional level, decreased COX (cytochrome c oxidase) enzyme activity, together with lower ATP content, was observed in *Mtm1*^−/y^ muscles [31]. In addition, decreased expression of oxidative phosphorylation genes and decreased COX activity was present in the *Mtm1* gene trap model [61]. Zebrafish with *ryr* reduction displayed an increase in mitochondrial oxidative stress, possibly due to excessive calcium influx from overloaded SR to mitochondria [151]. Of note, DNM2 was recently proposed to mediate mitochondrial fission together with DRP1 [188], although this point is still controversial [189,190]. Enlarged mitochondria lacking cristae were observed in the muscle from the skeletal muscle specific DNM2-KO mouse [95], and reducing the level of DNM2 in *Bin1*ex20^mck−/−^ and *Dnm2*-KI (R465W and S619L) mouse models rescued mitochondrial anomalies [94,135,191]. Altogether, this suggests that the balance of DNM2 activity/expression is important for mitochondria homeostasis and positioning, directly or indirectly. MTM1 was also described to have a direct, non-desmin-dependent role in mitochondrial distribution, dynamics, and function [31]. As previously discussed, cytoskeleton and, in particular, desmin may be implicated in mitochondrial positioning, as loss of desmin induces defects in mitochondrial position and dynamics [192].

### 6.3. Membrane Trafficking

MTM1, DNM2, and BIN1 are proteins involved in membrane trafficking and all three bind phosphoinositides at membranes. Moreover, MTM1 dephosphorylates PIs, modifying the physico-chemical properties of membranes and the recruitment of proteins, while BIN1 and DNM2 induce membrane curvature and potentially fission.

#### 6.3.1. Endocytosis

The function of dynamin in endocytosis was described for the first time with the characterization of the shibire gene (unique drosophila ortholog) implicated in synaptic vesicle recycling [193]. This role was more recently demonstrated in mammalian cells expressing dynamin mutants with reduced GTPase activity [194]. Dynamins catalyze membrane fission, promoting vesicle internalization. The recruitment of dynamin is dependent on interaction with SH3 containing proteins (such as amphiphysin 1, 2, and sorting nexin 9) through its PRD domain [195,196] and on binding to phosphoinositides such as PI (4,5)P2 through its PH domain [197]. The ability of dynamin to catalyze membrane fission is required for both clathrin mediated endocytosis (CME) and clathrin-independent endocytosis. The impact of CNM mutants in endocytosis was largely studied with contradictory findings, perhaps due to differences in experimental setup and cell lines. For example, COS cells overexpressing CNM mutants [180,198] and fibroblasts from R465W homozygous mice displayed impaired CME [93], while no effects were observed in patient and mouse DNM2^R465W/+^ fibroblasts [180]. A stable cell line was established from *Dnm2* knock-out mouse fibroblast expressing DNM2-CNM mutants to mimic the heterozygous and homozygous states. These CNM mutants fully rescued CME, even in the absence of WT-DNM2, although they impaired clathrin-independent endocytosis [199]. Transferrin (TfR) uptake was studied in myoblasts with endogenous levels of DNM2-R369Q and R465W mutations and in myoblasts from DNM2^R465W/+^ mice [114]. An increased CME was noted, in agreement with in vitro results showing the hyperactivity of CNM mutants. Further studies will be needed to assess the involvement of the endocytic pathway alteration in skeletal muscle and in the pathophysiology of CNM. As this pathway is not muscle specific, the relevance in other tissues should also be considered, although non-muscle tissues may depend more on the activity of other dynamins such as dynamin 1 in the brain. It has been also described that the internalization of glucose transporter GLUT4, specifically expressed in muscle and adipose tissue, is mainly mediated by DNM2-mediated endocytosis [200,201]. DNM2^R465W/+^ myofibers exhibited defective insulin induced GLUT4 translocation [111].

Concerning BIN1, its N-BAR domain binds membranes and promotes tubulation, a process necessary for endocytosis. Importantly, only the neuronal BIN1 isoform 1, and not the muscular BIN1 isoform 8, contains a CLAP domain that is able to bind clathrin and AP2 to mediate clathrin mediated endocytosis [117]. As knock-down of the ubiquitous BIN1 isoform, which does not contain the CLAP domain, did not affect transferrin uptake [201], a main impact of CNM mutations in BIN1 may not be on endocytosis, but probably on biogenesis of the T-tubule.

#### 6.3.2. Endosome Recycling

The PI desphosphorylation activity of MTM1 plays a crucial role in membrane recycling from endosomes to the plasma membrane, which is dependent on phosphoinositide conversion [35]. MTM1 depletion leads to PI(3)P accumulation in endosomes, resulting in the accumulation of signaling receptors such as epidermal growth factor receptor in these compartments [202]. MTM1 is also implicated in integrin recycling, which forms the major component of focal adhesions at costameres [73]. In 2011, Ribeiro et al. observed that depletion of the MTM1 drosophila ortholog *mtm* promoted the accumulation of integrins in endosomes [36]. This defect in integrin localization was later confirmed in XLMTM cells and human and mouse muscle sections [35,36,73]. More specifically, MTM1 is involved in β-1 integrin recycling from endosomes to the plasma membrane as this process requires phosphoinositide conversion from PI(3)P to PI(4)P [35]. The accumulation of β-1 integrin in early endosomes in *Mtm1*^−/y^ cells and myofibers correlates with focal adhesion defects, and reduction in the cell migration and myoblast fusion [73]. In muscle, this alteration in the costamere and link to the extracellular matrix due to defective integrin recycling may explain the decreased fiber size, rounded fiber shape and increased interfiber space found in XLMTM. These histological defects and β-1 integrin accumulation were efficiently rescued when BIN1 was overexpressed [73], suggesting that BIN1 and MTM1 are functionally linked and participate together in the maintenance of focal adhesions in skeletal muscle. Indeed, amphiphysins are also involved in endosome recycling. BIN1 was described to localize with endosome markers such as EEA1, APPL1, CD63, Rab11, and EHD1 [203,204] and the *C. elegans amph-1* (BIN1 ortholog) null mutant presented defective recycling endosome function and morphology [205]. BIN1 knock-out in HeLa and MEF cells did not impair transferrin uptake, however, recycling was impaired [132,205]. Less is known about the participation of DNM2 in endosome recycling in skeletal muscle. It was described to play a role in vesicle trafficking from the late endosome to Golgi compartment or in recycling pathways from early endosomes to the lysosome or plasma membrane in cultured cells [206]. Expression of the temperature sensitive mutant of DNM1 (dynamin 1^G273D^, *dyn*^ts^) in HeLa cells causes defects in transferring recycling [207]. Recently, DNM2 was shown to be a crucial player in a process relevant for muscle tissue: triggering the scission and release of autophagosome precursors from recycling endosomes [113], a critical step for autophagy described in more detail in the section on protein homeostasis.

### 6.4. Protein Homeostasis

Autophagy-lysosome, together with the ubiquitin-proteasome system (UPS), are the main catalytic pathways for protein degradation and organelle clearance. They coordinate in muscle to maintain protein homeostasis and its dysregulation has been shown to contribute to the pathogenesis of several neuromuscular disorders [208].

#### 6.4.1. Autophagy

Autophagy is a natural mechanism in which dysfunctional organelles or protein aggregates are engulfed in specific structures called autophagosomes that fuse with lysosomes for cargo degradation. The autophagic flux can be measured by the amount of proteins such as LC3-II (membrane protein at the autophagosome) and p62 (specifically degraded by autophagy) [209]. Defects in autophagy have been associated with numerous diseases including myopathies. For example, the defect in fusion of lysosome to the autophagosome caused by mutations in the lysosomal protein LAMP-2 induces the accumulation of autophagosomes and contributes to X-linked vacuolar cardiomyopathy and myopathy [210]. The maintenance of muscle mass depends on the balance between protein synthesis and degradation. As autophagy flux dysregulation has been shown to induce muscle atrophy [211], it is proposed to be a main cause of muscle atrophy in CNM where it correlates with the accumulation of mitochondria, membrane structures, the disorganization of sarcomere, and enlargement of SR.

Myotubularin deficiency results in increased PI(3)P levels, a key lipid for endosomal trafficking (as previously mentioned), and also participates in autophagosome formation and autophagosome-lysosome fusion [212,213]. PI(3)P levels were found to be elevated in skeletal muscle from *mtm1* morphant zebrafish [34], *Mtm1* deficient and *Mtm1* knock-in mouse models [60,61,63], and myotubes from XLMTM patients [29]. *Mtm1* deficiency in mice results in defective autophagy with the accumulation of p62 aggregates and abnormal mitochondria together with mTORC1 overactivation (negative regulator of autophagy) [66]. Interestingly, this defect in autophagy was mainly detected in skeletal muscle tissues, thus contributing to the skeletal muscle-specificity of the disease [61]. Defective autophagy with the accumulation of autophagic vesicles was also found in MTM1-Q384P dogs [65]. Normalization of autophagy by either re-expression of *MTM1* or the inactivation of the mTORC1 rescued muscle mass in *Mtm1*^−/y^ mice [61,66]. Thus, the autophagy defects may also contribute to muscle dysfunction due to the accumulation of dysfunctional mitochondria, ubiquitinated proteins, and aggregation of proteins such as desmin. Moreover, the early increased expression in genes related to atrophy, commonly named as atrogenes, may play a key role in the onset of the severe muscle atrophy in *Mtm1*^−/y^ mice [66]. It can be hypothesized that at the early stage of the disease, the high PI(3)P levels will activate autophagy, while also blocking autophagosome-lysosome fusion necessary for protein clearance [212]. At later stage autophagy, inhibition would be exacerbated by mTORC1 signaling. The relationship between MTM1 and mTORC1 is still not clear, but some studies have shown that PI(3)P levels may modulate mTORC1 activity [214].

Fiber hypotrophy has also been described in mouse models with DNM2 mutations [93,108]. This hypotrophy could be linked to an imbalance of anabolic/catabolic pathways as, similar to *Mtm1*^−/y^ mice, transcriptional activation of the ubiquitin-proteasome and autophagy pathways was found at two months in the *Dnm2*^R465W/+^ mice [93,107]. Fasting *Dnm2*^R465W/+^ mice led to an accumulation of immature autophagic structures [107]. An autophagy impairment in basal conditions was observed in *Dnm2*^R465W/+^ immortalized mouse myoblasts, and rescued with allele-specific correction of this mutation by CRISPR/Cas9 [114]. The fact that *Dnm2* expression is induced in cells under starvation conditions suggests a key implication of DNM2 in the autophagic process [107]. Indeed, the role of DNM2 in autophagic lysosome reformation (ALR) was described in 2013 in hepatocytes [215]. ALR is a process that aims to maintain lysosome homeostasis by generating new functional lysosomes from autolysosomes, where DNM2 may normally mediate the proto-lysosome scission [216]. Defective ALR have recently been associated with the cause of skeletal muscle disease [217]. DNM2 was also found to locate specifically on recycling endosomes where it binds LC3 and regulates the release of autophagosome precursors. This process is impaired with the R465W mutation, which sequesters DNM2 at the plasma membrane where it binds ISTN1 at endocytic sites [113]. BIN1 mutations and deficiency may also affect the autophagy pathway, as increased p62 staining was described in BIN1-related ADCNM muscles [5]. In addition, ultrastructural studies in *Bin1e*x20^mck−/−^ mice revealed the accumulation of autophagosomes confirmed by LC3 staining in muscle sections [135]. Concerning RYR1, increased LC3II and p62 protein levels, together with mTORC1 overactivation and inhibition of autophagy flux, were observed in the *Ryr1-Rec* mice [43]. Dysregulation of cytoplasmic calcium may affect autophagy [218]. DHPR downregulation in mice has also been reported to cause autophagy abnormalities [219].

#### 6.4.2. Ubiquitin-Proteasome System

The ubiquitin-proteasome system (UPS) is also an important catalytic pathway involved in the clearance of unfolded and misfolding proteins. A direct link was described between MTM1 and UPS. MTM1 binds to UBQLN2, a protein that associates with both proteasomes and ubiquitin ligases [220], and mediates the proteasomal degradation of misfolded intermediate filaments such as desmin and vimentin [69]. *Mtm1* KD in cells and mouse muscles causes the accumulation of desmin aggregates and poly-ubiquitinated conjugates as well as the activation of unfolded protein response (UPR). These defects were restored when MTM1-WT and a MTM1 phosphatase-dead mutant were expressed, while overexpression of the MTM1-K255A mutant, which abolishes binding to UBQLN2, did not restore these phenotypes [69].

As previously described, the DNM2-R465W mutant promotes autophagy defects in muscle fibers and cells, whereas the proteasome activity was found to be normal, together with unchanged levels of ubiquitinated proteins in MEF cells homozygous for that mutation [107]. However, it was recently proposed that DNM2 is degraded by the UPS in an ERα-dependent manner [221]. ERα is an estrogen receptor found in muscle and has been described to mediate the activation of UPS [222]. Tamoxifen may act as an agonist of ERα in muscle [221]. Activation of UPS or treatment with tamoxifen was found to decrease DNM2 protein levels in the context of *Mtm1* deficiency [221]. Thus, defective UPS in *Mtm1*-deficient muscle would lead to the accumulation of cytoskeletal proteins and may contribute to the increased DNM2 expression observed in XLMTM.

While there is no reported link between protein homeostasis and BIN1, calcium (which is modulated by RyR1) has been described to directly induce muscle proteasome activity [223].

### 6.5. The Neuromuscular Junction

The signal triggering muscle contraction originates in the nervous system and is transmitted to the muscle at the neuromuscular junction (NMJ), which is a synaptic connection between the terminal end of a motor neuron and a muscle fiber.

There are some cases where CNM patients have been misdiagnosed with congenital myasthenic syndrome because they had abnormal neuromuscular transmission in neurophysiology studies. However, a diagnosis of CNM was suggested by histopathology. They presented fatigability, which favorably responded to acetyl cholinesterase (AChE) inhibitors (pyridostigmine) [224]. AChEs are enzymes responsible for the degradation of the acetylcholine neurotransmitter, so its inhibition will increase the duration of action of acetylcholine. These findings led to the study of NMJ in the *mtm1* morphant zebrafish, showing defects in NMJ organization and improvement in motor function in response to the AChE inhibitor edrophonium [224]. A defective NMJ structure has also been observed in autosomal CNM and XLMTM patients [225,226,227]. NMJ defects and the potential of AChE inhibitors were further investigated in the *Mtm1^−/y^* mouse model [68]. NMJs of *Mtm1*^−/y^ mice were more disorganized than in the control muscle and structurally abnormal, with increased size and lower complexity. Ultrastructural analysis revealed disorganized sarcoplasm at the post-synaptic junction, with increased vacuolar content that may be due to defective endosome trafficking at/to the NMJ. Dysfunction of NMJ transmission was also described in the *Mtm1^R69C/y^* mouse with amelioration after pyridostigmine treatment [68].

Defects in NMJ transmission may also play a role in the pathogenesis of DNM2-CNM. A disorganized NMJ pattern was observed in a zebrafish model transiently overexpressing the human DNM2-S619L mutant, and treatment with AChE inhibitors rapidly alleviated the motor dysfunction observed in these animals [39]. Additionally, evidence of abnormal neuromuscular transmission was found in five patients with DNM2 mutations (S619L and E368K), with favorable response to pyridostigmine treatment. Accordingly, defects in NMJ number and fragmentation were found after intramuscular overexpression of DNM2-WT or DNM2-CNM mutants in mice, correlating with the decrease in muscle force observed [98]. Gain-of-function but also loss-of-function of DNM2 may cause defects in NMJ as a specific deletion of *Dnm2* in skeletal muscle promotes irregular NMJs, resulting in degenerative intramuscular peripheral nerves [95]. However, no obvious NMJ defects were reported in *Dnm2*^S619L/+^ and *Dnm2*^R465W/+^ mouse models [93,94], although further studies will be required. Dynamin is known to regulate synaptic vesicle recycling at the presynaptic membrane [83]. Recently, DNM2 was shown to regulate postsynaptic development of NMJ via actin remodeling at the structures known as synaptic podosomes in mouse myotubes [228]. In this study, DNM2 was able to oligomerize around actin filaments forming bundles to regulate podosome maturation and turnover. CNM mutants affected this process and were linked with defects in the electrophysiological activity of drosophila NMJ [228].

Amphiphysin is also localized post-synaptically at the NMJ in drosophila larvae [155]. However, no defects in neurotransmission were found in the drosophila *amph* mutant, supporting that this protein is not essential for the development and synaptic vesicle recycling at NMJ, at least in flies [155]. It would be interesting to check whether BIN1 deficiency has any impact on NMJ formation and maintenance in other models and in CNM patients. We can expect to observe some defects as transcriptomic analysis of *Dnm2*^S619L/+^, BIN1ex20^mck−/−^, and *Mtm1*^−/y^ mice showed upregulation in the expression of acetylcholine receptor subunits (*Chrna1*, *Chrna9*, *Chrnd*) [153]. Dysregulation of these genes was also observed in XLMTM patients [229] and *MTM1* dogs [154].

No role for RyR1 at the postsynaptic compartment of the NMJ has been proposed as of yet, and no NMJ defects were observed in the *ryr1* relatively relaxed zebrafish model [150]. However, several studies have documented a role for RyR1 in presynaptic release at the NMJ [230,231].

### 6.6. Muscle Regeneration

Satellite cells (SCs) are resident stem cells of the muscle providing a source of nuclei for muscle growth and regeneration [232]. In response to injury, they proliferate intensely to repair the damaged tissue [233]. A decrease in *Pax7*+ SCs progressing with age was described in *Mtm1^−/y^* myofibers [72]. *Mtm1^−/y^* myoblasts displayed increased apoptosis, lower proliferation, and poor engraftment, resulting in a lower number of dividing cells and SCs during regeneration [72]. Fewer SCs were also detected in gastrocnemius and quadriceps from *Mtm1*^R69C/y^ mice [74]. Similarly, SCs were significantly lower in patients with MTM1 deficiency compared to the controls [32]. Inhibition of myostatin increases the number of SCs in *Mtm1*^R69C/y^ gastrocnemius [74]. Similarly, a decrease in SC content has been found in *Dnm2*^R465W/+^ muscles [108]. Almeida et al. investigated the consequences of SC depletion in *Dnm2*^R465W/+^ muscles and found that muscle regeneration was less efficient and slower in *Dnm2*^R465W/+^ mice [106]. The *Dnm2* mutation affects SC proliferation capacity and activation, with altered expression of myogenic factors in response to muscle injury. SC content and function was not studied in *Bin1e*x20^mck−/−^ muscles. However, it has been investigated in *Bin1*ex11^−/−^ mice, which displayed a slower regeneration after muscle damage, potentially due to the reduced pool of SCs found in this mouse [131]. A defective muscle regeneration pathway was also recently suggested from the common CNM transcriptomic signature (*Mtm1*^−/y^, *Dnm2*^R465W/+^ and *Bin1e*x20^mck−/−^)[153] and may be related to the observed activation of the inflammatory pathway [234]. Further studies will be needed to determine the specific mechanism implicating the CNM genes in SC survival and functions.

## 7. Therapeutic Targets in CNM

In this section, we discuss an update of the potential therapies that may be applicable to treat one or more forms of CNM (Table 6). A more detailed description of most therapies tested in preclinical studies and a comparison between them can be found in the review by Tasfaout et al. [235].

### 7.1. Common Therapeutic Strategies

- Gene silencing: DNM2 reduction or normalization

The aim of this approach is to normalize or reduce DNM2 protein levels to compensate for the increase in DNM2 expression or activity in different CNM forms.

In vitro experiments have shown that DNM2-CNM mutations promote oligomerization and result in increased GTPase activity [77,78,79]. Overexpression of human WT DNM2 in mouse muscle induced CNM phenotypes [97,98] and its overexpression in other systems such as zebrafish, drosophila, or mouse myoblasts also led to muscle CNM phenotypes and excessive T-tubule fragmentation [77,102]. Altogether, these data support that DNM2-CNM mutations are gain-of-function. In addition, muscle biopsies from *Mtm1*^−/y^ mice and XLMTM patients displayed an increase of DNM2 protein. Altogether, these observations led to a ‘cross-therapy’ strategy whereby decreasing DNM2 expression was tested in CNM models linked to mutations in different CNM genes. [96]. A first proof-of-concept was achieved by genetic crossing of *Mtm1*^−/y^ with *Dnm2*^+/−^ mice and obtention of *Mtm1*^−/y^ *Dnm2*^−/+^ males with rescued muscle histology, muscle force, and locomotor activity compared to *Mtm1*^−/y^ littermates [96]. Decrease or normalization of the total DNM2 levels could also be achieved by either AAV-mediated expression of shRNA targeting *Dnm2* mRNA or using antisense oligonucleotides (ASO) against *Dnm2* pre-mRNA and mRNA, which ameliorated the *Mtm1*^−/y^ phenotypes [184,236]. Systemic ASO delivery resulted in prevention and even reversion of the CNM phenotypes [236]. The concept of cross-therapy in CNM was then further expanded to other CNM forms. *Bin1e*x20^−/−^ mice normally die on the first day of life, whereas genetic reduction in DNM2 levels to 50% resulted in viable pups with normal survival (analyzed up to 18 months), muscle force, and triads [84]. Acute DNM2 downregulation with *Dnm2* ASO was recently tested in the viable BIN1 mouse model (*Bin1e*x20^mck−/−^), which displayed a slight increase in DNM2 protein level; DNM2 reduction improved muscle phenotypes with total correction of mitochondrial mislocalization [135]. Finally, ASO systemic injections positively rescued CNM phenotypes in the moderate *Dnm2*^R465W/+^ and severe *Dnm2*^S619L/+^ mouse models, respectively [94,191]. Of note, *Dnm2*^S619L/+^ mouse muscles also presented increased DNM2 protein expression when untreated. In addition to providing a potential common therapeutic approach, these findings validate the epistasis between MTM1, BIN1, and DNM2.

Dynacure is a company leading further preclinical development of an ASO product targeting *DNM2* (www.dynacure.com). After confirmation of safety and tolerability in preclinical models and non-human primates, the UNITE-CNM trial was launched as a phase 1/2 study (ClinicalTrials.gov Identifier: NCT04033159). This study aims to assess safety, tolerability, pharmacokinetics (PK) and pharmacodynamics (PD) as well as the preliminary efficacy of intravenous injection (IV) of an ASO against human *DNM2* mRNA in male and female patients ≥16 years with confirmed *DNM2* or *MTM1* mutations. Another study is also planned in clinically symptomatic pediatric patients from two to 17 years (DyNaMic study, ClinicalTrials.gov Identifier: NCT04743557), pending the results of the first trial.

- Acetylcholinesterase inhibition

The rationale of this therapy is to improve the poor NMJ transmission observed in XLMTM and DNM2-CNM patients and animal models. The acetylcholinesterase inhibitors edrophonium (zebrafish) and pyridostigmine (mice and human) were tested. These drugs prevent the degradation of the acetylcholine neurotransmitter, prolonging its half-life at the NMJ. They were tested in *mtm1* morphant zebrafish [224], zebrafish with transient expression of the DNM2-S619L mutation [39], and *Mtm1*^R69C/y^ mice [68], leading to motor function improvements. Pyridostigmine is a drug already approved by the FDA and used in patients to treat myasthenia gravis [250]. Pyridostigmine treatment in patients with DNM2 mutations resulted in reduced fatigability and improvement in muscle strength and motor function [39]. Acetylcholinesterase inhibitors also resulted in favorable responses in XLMTM patients [224].

### 7.2. Specific Therapeutic Strategies

- Gene replacement: MTM1 re-expression in XLMTM

The rational of this approach is to express MTM1 using AAV vectors in XLMTM patients as most patients lack MTM1 protein expression [19,20,238]. The preclinical studies were conducted on *Mtm1*^−/y^ mice and XLMTM dogs. In *Mtm1*^−/y^ mice, AAV-mediated *Mtm1* intramuscular expression under the control of the ubiquitous CMV promoter resulted in the recovery of muscle mass, force, and histology [239]. Then, mice and dogs lacking MTM1 were treated with a single systemic or locoregional injection of AAV-*Mtm1* (or canine *MTM1*) under the control of the desmin promoter for specific muscle expression [238,240,251]. Successful results were achieved, with muscle phenotypes rescued together with increase in lifespan and body weight. No safety issues were observed in dogs, in which long-term expression of myotubularin four years after injection was shown, corresponding with gait and respiratory functions comparable to age-matched healthy control dogs [251]. This therapy was then translated to human clinical studies after testing safety in non-human primates with tolerated doses up to 8 × 10^14^ vg/kg [252]. The initiation of a clinical trial for AAV-MTM1 gene therapy took place in 2017 by Audentes Therapeutics (acquired by Astellas Pharma in 2019, www.astellasgenetherapies.com). The ASPIRO Phase 1/2 Study (ClinicalTrials.gov Identifier NCT03199469) aims to evaluate the safety and efficacy of AT132 (resamirigene bilparvovec; rAAV-Des-h*MTM1*) in XLMTM male patients younger than five years old with mechanical ventilatory support. The first part of the study consists of a dose escalation phase where doses of 1.3 × 10^14^ vg/kg and 3.5 × 10^14^ vg/kg are tested, followed by a pivotal expansion to further confirm the efficacy and safety of the highest dose. Striking preliminary results showing the therapeutic efficacy of the study were presented in October 2019, with a robust expression of MTM1, 24 and 48 weeks after the single injection of AT132 [253]. Muscle biopsies showed improvements in fiber size and organelle mislocalization compared to baseline, although persistence of internal nuclei in many samples was observed. Significant improvements in neuromuscular function and respiratory strength were quantified, with some patients even able to sit or walk without any support or acquiring total ventilator independence. However, after this release, three patients treated with the higher dose and one patient treated with the low dose presented with hepatobiliary complications a few weeks after dosing and later died, likely due to treatment-related complications currently under study [254]. The three study participants in the high dose cohort had clinical evidence of cholestasis before dosing [252,254]. Recent studies suggest a subset of XLMTM patients may present with underlying hepatobiliary disease, and in particular, intermittent presentation of cholestasis [24,25].

- Enzyme replacement: MTM1 delivery in XLMTM

Similar to the rationale for gene replacement, here, the strategy is to directly replace the missing enzyme. It is based on the delivery of MTM1 fused to a mouse monoclonal antibody (3E10Fv) designed to increase uptake into muscle fibers (3E10Fv-MTM1 fusion protein). Preclinical studies were conducted on *Mtm1*^−/y^ mice by intramuscular injection of four doses of this fusion protein, with slight improvement in muscle force potentially linked with increased number of triads and T-tubules found after treatment [241]. Further development of this product was announced by Valerion (VAL-0620, www.valerion.com), which uses the 3E10 antibody technology to replace enzymes in other disorders such as Pompe disease.

- Gene transfer: MTMR2 expression in XLMTM

MTMR2 is a ubiquitously expressed homolog of MTM1 (65% amino acid sequence identity) with phosphatase activity [255]. Intramuscular and systemic injections of AAV expressing *MTMR2* in *Mtm1*^−/y^ showed amelioration of all muscle phenotypes together with the normalization of PI(3)P levels [242,243]. In addition, a short isoform of MTMR2 that provides a better rescue was identified [243]. MTMR2 gene therapy may therefore be a possible alternative to MTM1 re-expression in XLMTM to restore PI(3)P levels while bypassing potential immunoreactivity toward MTM1 re-expression.

- Gene transfer delivery: BIN1 expression in XLMTM

Another gene therapy alternative was recently described by Lionello et al. based on the expression of *BIN1* in the *Mtm1*^−/y^ mouse [73]. The genetic and AAV-mediated overexpression of human *BIN1* rescued lifespan and improved muscle force and histological defects. It also led to a striking rescue of the *Mtm1*^−/y^ muscle transcriptome [153]. As with DNM2 downregulation, this study strengthens the “cross-therapy” rationale, where the modulation of one CNM gene (*BIN1*) can rescue the loss of another CNM gene (*MTM1*). BIN1 is a known interactor of MTM1 in skeletal muscle and MTM1 was described as a positive regulator of BIN1 membrane tubulation properties. Both are implicated in endosome recycling as previously described, and BIN1 expression was shown to rescue recycling of β1-integrin to [130] the plasma membrane [73]. In addition, BIN1 is a known interactor of DNM2 and several studies have shown that it can negatively regulate DNM2 activity in vitro [84,161].

- Allele-specific targeting of DNM2 mutations in DNM2-related ADCNM

Different approaches have been proposed to specifically target the transcript containing *DNM2* mutations. The first approach, named spliceosome-mediated RNA trans-splicing (SMarT), consists in the correction of the mutation at the post-transcriptional level by modifying the mRNA sequence [256]. It was tested in vitro in fibroblast and in vivo in WT muscle and results in successful trans-splicing events [257]. A second strategy, allele-specific inactivation or correction of the heterozygous DNM2 R465W mutation directly in the DNA, was successfully achieve by CRISPR/Cas 9 in the patients’ fibroblasts and *Dnm2*^R465W/+^ mouse myoblasts [114]. This approach rescued autophagy and transferrin uptake defects observed in the mutant mouse myoblasts. Finally, allele-specific RNA silencing has been achieved in patient-derived fibroblast and in DNM2^R465W/+^ mice [237]. This approach is based on the design and validation of siRNA to specifically silence the mutated DNM2 transcript without affecting the WT transcript. This approach resulted in rescued muscle mass, specific force, and histology in young DNM2^R465W/+^ mice. For these different approaches, potential toxicities, and off-targets, and sustained in vivo efficacy will have to be carefully investigated.

- Exon skipping for RYR1-related myopathy

A first proof-of-concept for exon skipping was carried out on cells from a patient with recessive RYR1 mutations associated with a congenital myopathy [244]. In this case, a deep intronic mutation results in the exonization of an intronic sequence, leading to a destabilized and decreased protein. U7-antisense oligonucleotides expressed from a lentiviral vector successfully allows for the skipping of the pseudo-exon in primary muscle cells, leading to an increase in the RYR1 protein and the restoration of calcium release of normal amplitude.

- Cell transplantation or cell therapy in XLMTM

Cell transplantation aims to compensate the satellite cell depletion observed in different CNM animal models and patients [32,72,74,106,108,131]. Moreover, satellite cells from *Mtm1*^−/y^ mice were defective and unable to “rebuild” damaged skeletal muscle. A cell therapy approach was tested in the mild *Mtm1^R69C/y^* mouse model by the injection of skeletal muscle-derived WT myoblasts into the gastrocnemius [245]. This resulted in slight improvements in muscle strength and mass.

- Myostatin inhibition in XLMTM

This approach was designed to counteract hypotrophic signaling pathways in *Mtm1*^−/y^ mice. The product used for preclinical studies consisted of the soluble form of the myostatin receptor, activin type II receptor (ActRIIB-mFC) [246]. In *Mtm1*^−/y^ mice, the inhibition of myostatin action results in a small prolongation of lifespan (17%) and an increase in muscle weight with a slight and transient improvement in forelimb grip strength correlated with hypertrophy of type 2b fibers [246]. The same approach was tested in the less severe *Mtm1*^R69C/y^ mouse model with no clinical benefit, resulting in increased gastrocnemius weight but not quadricep weight [74]. This was potentially explained by the increase in satellite cell activation after treatment only in the gastrocnemius [74]. Myostatin inhibitors were also shown to be beneficial in DMD mouse models, but this positive impact has not been later translated in clinical studies [258]. Potentially, this low benefit in XLMTM could be due to the low myostatin plasma levels found in sick *Mtm1*^−/y^ mice and observed in XLMTM and ADCNM patients [259,260].

- Pharmacologic inhibition of mTORC1 in XLMTM

In this case, mTORC1, a main negative regulator of autophagy, was targeted to restore autophagy defects in the *Mtm1^gt^*^/*y*^ mouse model. Mice were treated for three days with oral administration of AZD8055, an ATP-competitive inhibitor of mTORC1 activity [61]. The treatment induces autophagy-mediated degradation in *Mtm1* deficient muscles and results in the improvement of the muscle mass. This drug is already used in humans to treat cancer and prevent organ transplant rejection [261].

- Pharmacological inhibition of PI3K in XLMTM

PI3K inhibition aims to rebalance the elevated PI(3)P levels in muscles with myotubularin deficiency [34,60,63,158]. In mice, the class II kinase PIK3C2B was shown to be a modifier of *Mtm1*^−/y^ disease [63]. Its total genetic ablation in this mouse model resulted in a striking rescue of survival and muscle phenotypes, while partial PIK3C2B reduction had a milder impact. Conversely, the reduction in the class III kinase PIK3C3 worsened the muscle phenotypes. Wortmannin, a general PI3K inhibitor, ameliorated muscle phenotypes and slightly prolonged survival in *mtm1* zebrafish and *Mtm1*^−/y^ mice [63,70], and rescued calcium release defects in isolated *Mtm1*^−/y^ myofibers [70]. However, the achieved amelioration was not complete, most likely because this drug is not specific for PIK3C2B and also targets PIK3C3. Specific inhibitors for class I and III PI3K did not show any rescue in *mtm1* deficient zebrafish. Thus, the challenge of this therapy will be to develop specific and potent PIK3C2B inhibitors. Of note, expression of phosphatase-death MTM1 in *Mtm1*^−/y^ myofibers rescued its intracellular structure and improved the muscle performance, pointing to phenotypes independent of PI(3)P levels [183].

- Drug repurposing: Pharmacologic inhibition of p38 MAPK in RYR1-related AR myopathy

Several FDA-approved inhibitors of p38 MAPK were identified as potential therapeutic candidates for RYR1-related myopathies using a multi-species discovery pipeline [247]. First, these potential hits were uncovered in a screening conducted using the *C. elegans* RyR model [262]. However, none of those compounds were able to improve movement in the *ryr1b* single or *ryr1a/ryr1b* double mutant zebrafish model. These drugs were tested in muscle cells with opposing results depending on the RyR1 level: they inhibited calcium release in normal conditions, but they also enhanced calcium release in the absence of RyR1 [247]. However, the mechanism of action of p38 inhibitors needs to be further investigated and its efficacy should be tested in novel mouse models with a low RyR1 amount and/or RyR1 loss-of-function.

- Drug repurposing: antioxidant therapy in RYR1-related AR myopathy

The antioxidant N-acetylcysteine (NAC) was described to restore the motor function and improve the histology in the *ryr relatively relaxed* zebrafish mouse model [151]. This finding was confirmed in myotubes derived from RYR1-related myopathy patients, where this antioxidant reduces cell death induced by the oxidant H_2_O_2_ [151]. This treatment aims to counteract the downstream effects of calcium dysregulation on the mitochondria. NAC is a FDA-approved drug with a limited side effect profile. As no specific treatments exist for RYR1-related myopathies, it was decided to test whether NAC could decrease oxidative stress and increase muscle endurance in those patients in a phase1/2 clinical study (ClinicalTrials.gov Identifier: NCT02362425). The results of this study, initiated in 2015, were recently published, showing that NAC was unable to decrease the elevated oxidative stress in patients with no improvements in exercise tolerance [248].

- Drug repurposing: tamoxifen treatment in XLMTM

Tamoxifen is a selective estrogen receptor modulator that has been used in breast cancer treatment since the 1970s. It is an FDA-approved drug with a good safety profile including in pediatric patients. In 2013, Dorchies et al. used this drug to treat a Duchenne muscular dystrophy (DMD) mouse model, showing an anti-fibrotic effect in the heart and diaphragm as well as muscle force improvements [263,264]. This led to the initiation of a Phase 1 (ClinicalTrials.gov Identifier NCT02835079) and later a Phase 3 clinical trial on DMD boys (ClinicalTrials.gov Identifier NCT03354039) to test the safety and efficacy of tamoxifen to reduce disease progression [265]. Then, in 2018, two parallel independent studies showed the efficacy of tamoxifen treatment to prolong survival and improve muscle force and histology of *Mtm1*^−/y^ mice [221,249]. The exact mechanism of therapeutic rescue is not yet known, but it is not thought to be mediated by transcriptional modulation via an estrogen receptor as minimal transcriptional changes were observed after tamoxifen treatment [153,221]. Both studies showed normalization of the DNM2 protein levels, and as described in the UPS section, tamoxifen was proposed as a possible modulator of the DNM2 protein levels [221]. These positive data resulted in the initiation of a clinical trial, Phase 1/2, in male patients with *MTM1* mutations older than two years (TAM4MTM, ClinicalTrials.gov Identifier NCT04915846). The objective is to test the efficacy and safety of tamoxifen therapy to improve respiratory and motor function in XLMTM males.

## 8. Conclusions and Perspectives

In the last few years, several advances in the understanding of the pathomechanisms have been made, which have led to the identification of novel therapeutic strategies, some of which are now in clinical trials. Current therapeutic development is mainly focused on the XLMTM form, although the DNM2 targeting approach was shown to be potentially applicable to other CNM forms; DNM2 and BIN1-related CNM. More therapeutic strategies have been tested in XLMTM models as this was the first form described, however, now there are faithful animal models available for all CNM forms including recessive RYR1-CNM. These laboratory models will allow for a better investigation of disease mechanisms in those CNM forms and drug screening. Potentially several altered pathways can be targeted, suggesting the potential for combined therapies to treat CNM. This will need to be further investigated over the next years.

It is still unclear why mutations in these proteins, most ubiquitously expressed except for RyR1, cause predominantly tissue-specific phenotypes generally restricted to skeletal muscle. This may be due to the implication of these proteins in muscle specific structures such as costameres, NMJs, and triads. One of the most common pathomechanisms found in CNM with mutations in *RYR1*, *MTM1*, *DNM2*, and *BIN1* is a defective excitation–contraction coupling. This mechanism may be the main one underlying muscle weakness in CNM. Abnormal myonuclei and mitochondria position may be caused by cytoskeleton defects, while muscle atrophy might be explained by the dysregulation of protein homeostasis, transcription, and decrease in satellite cells. However, some non-muscle phenotypes have been described such as peripheral nerve involvement in some DNM2-CNM cases or liver dysfunction in XLMTM patients, calling for more investigation into non-muscle tissues.

## Figures and Tables

**Table 1:** Phenotype in patients with *MTM1*, *BIN1*, *DNM2*, or *RYR1* mutations.

CNM Form/ Mutated Gene	Incidence/ Prevalence [9,21]	Severity/ Age of Onset	Clinical Presentations (Common Findings) [1,21]	Muscle Histology [21,22]	Altered Pathways	Ref(s)
**XLMTM/** **MTM1**	17 per mln births */ 57% CNM patients	+++/ Neonatal,20% moderate late-onset form	**Severe** neonatal hypotonia, generalized muscle weakness. 25% male die in the first year of life. In late-onset cases, slowly progressive weakness. Moderate ptosis and ophthalmoplegia. Respiratory failure and swallowing difficulties. Others: **dolichocephaly**, possible hepatobiliary disease	Fiber hypotrophy, rounded fibers, centralized nuclei, type 1 fiber predominance. **Pale peripheral halo** lacking of oxidative activity. Female and late-onset cases: necklaces and internalized nuclei.	Abnormal triads Satellite cell deficiency NMJ dysfunction Increased levels of PI(3)P Epigenetic dysregulation Defective autophagy Defective endosome recycling	[3,26,29,30,31,32,33,34,35,36]
**ADCNM/** **DNM2**	2 per mln births */ 12% CNM patients	+ or ++/ Adulthood or adolescence, neonatal	Slowly progressive muscle weakness. Pediatric cases: generalized muscle weakness, hypotonia and breathing difficulties, improving over the time. Ophthalmoplegia and ptosis frequently present. Other: mild peripheral nerve involvement in some cases.	Hypotrophy of type 1 fibers, with some hypertrophic fibers, centralized or internalized nuclei, type 1 fiber predominance. **Radiating sarcoplasmic strands**, accumulation of oxidative activity around centralized nuclei.	Abnormal triad Defective autophagy NMJ dysfunction	[4,33,37,38,39]
**ARCNM/** **BIN1**	1 per mln births */ 4% CNM patients	++/ Childhood	Diffuse muscle weakness from slowly to rapidly progressive and facial weakness. Ophthalmoplegia and ptosis (some cases).	Fiber hypotrophy, rounded fibers, centralized and **clustered nuclei**, type 1 fiber predominance. Central accumulation of oxidative activity.	Abnormal triads Abnormal nuclear shape	[6,33,40,41]
**ADCNM/** **BIN1**		+/ Adulthood	Mildly progressive muscle weakness without facial involvement		Abnormal triads Defective autophagy	[5]
**ARCNM/** **RYR1**	2 per mln births*/ 12% CNM patients	++/ Neonatal, childhood	Proximal muscle weakness and hypotonia, improving over time. Ophthalmoplegia with or without ptosis. Possible breathing difficulties.	Fiber hypotrophy with size heterogenicity, predominantly **internalized** nuclei (some centralized), type 1 fiber predominancy. **Cores** depleted of oxidative activity with undefined boundaries.	Defective calcium homeostasis Epigenetic dysregulation Oxidative stress	[7,8,42,43,44]

* Incidence is expressed as affected newborns per million (mln) births. It represents the overall cases estimated for Europe, the U.S., Japan, and Australia [9]. Particularities in clinical presentations or histological findings that differentiate from other CNM forms are highlighted in bold.

**Table 2:** Main fish and mammalian models for XLMTM.

Genotype and Specie	Skeletal Muscle Phenotypes		Altered Pathways in Muscle	Ref(s)
	Lifespan and Motor Phenotype	Muscle Histology		
***Mtm1*^−/y^** **(*Mtm1*KO, *Mtm1*δ4) mouse**	Short lifespan (6–8 weeks), decreased body weight and progressive and generalized severe myopathy (from 3 weeks) with breathing difficulties.	Fiber hypotrophy, centralized and internalized nuclei, pale peripheral halo in oxidative staining.	Abnormal triads and defective ECC Deficient autophagy and UPS Dysfunctional mitochondria Abnormal NMJ Satellite cell deficiency and defective regeneration Increased levels of PI(3)P Defective endosome recycling	[31,35,59,63,66,67,68,69,70,71,72,73]
***Mtm1^gt^*^/*y*^** ***(gene trap)*** **mouse**	Short lifespan (6 weeks), decreased body weight and progressive and generalized severe myopathy (from 3 weeks).	Fiber hypotrophy, centralized and internalized nuclei.	Defective autophagy mTORC1 overactivation Dysfunctional mitochondria Increased levels of PI(3)P	[61]
***Mtm1*^Δ^^5/y^*, Mtm1*^Δ^^7/y^** mouse	Short lifespan (6–7 weeks) decreased body weight and generalized severe myopathy (from 3wks).	Fiber hypotrophy, centralized nuclei.	Defective muscular postnatal development and muscle maturation	[62]
***Mtm1*^R69C/y^** **(*Mtm1*-KI) mouse**	Reduced lifespan (median 66 weeks), non-progressive mild myopathy (from 8 weeks) and altered breathing.	Fiber hypotrophy, centralized nuclei. Central and peripheral accumulations of oxidative staining.	Abnormal triads NMJ dysfunction Satellite cell deficiency	[60,68,74]
***mtm1*** **morphant zebrafish**	Impaired motor function from 24 hpf.	Fiber hypotrophy, abnormal nuclei position and shape.	Abnormal triads and ECC Abnormal mitochondria (disrupted cristae) Increased levels of PI(3)P	[34]
***mtm1*** **-null (*mtm1*^Δ8/Δ8^) zebrafish**	Reduced lifespan (7–9 dpf) and impaired motor function and phenotypic changes from 3 dpf. Enlarged, globular and fatty liver.	Not specified.	Abnormal triads	[63]
***MTM1*** **N155K, Q384P dog**	Reduced lifespan, generalized and progressive severe myopathy (from 2–3 month).	Fiber size variability, hypotrophy type 1 fibers, centralized nuclei, type 1 fiber predominance. Subsarcolemmal and central accumulations of oxidative staining.	Abnormal triads Increased levels of PI(3)P Defective autophagy with Q384P mutation Transcriptional dysregulation with N155K	[64,65,75]

Hpf: hours post fertilization, dpf: days post fertilization, ECC: excitation–contraction coupling, NMJ: neuromuscular junction, PI(3)P: phosphatidylinositol 3-phosphate.

**Table 3:** Main fish and mammalian models for DNM2-related ADCNM.

Genotype and Specie		Skeletal Muscle Phenotypes		Altered Pathways in Muscle	Ref(s)
		Lifespan and Motor Phenotypes	Muscle Histology		
***Dnm2*^R465W/+^** mouse (mild adult CNM form)		Homozygous: died at P1 (2% survived 3 weeks). Heterozygous: normal lifespan, normal body weight, progressive moderate myopathy, normal body weight. Progressive moderate myopathy (from 3 weeks).	Fiber hypotrophy, normal nuclei position, central accumulation of oxidative activity.	Defective ECC and calcium homeostasis Defective autophagy Defective actin organization and polymerization Defective GLUT4 trafficking Defective costamere Deficient satellite cells	[93,106,107,108,109,110,111,112,113,114]
***Dnm2*^S619L/+^** (*Dnm2*^SL/+^) mouse (severe neonatal CNM form)		Homozygous: none survived to P2. Heterozygous: partial mortality from E18.5 to P10. Normal lifespan after P10, decreased body weight, generalized severe myopathy.	Fiber hypotrophy, normal nuclei position, central accumulation of oxidative activity.	Abnormal mitochondria (swollen and disrupted cristae)	[94]
**Transient expression of *DNM2***	Mouse h*DNM2*-WT, R465W, S619L	Decreased muscle force 2- and 4-weeks post-injection (higher impact of mutants).	Fiber hypotrophy, centralized and internalized nuclei Central and peripheral accumulations of oxidative activity.	Abnormal triads Abnormal mitochondria (enlarge and disrupted cristae with S619L mutant) Abnormal NMJ	[97,98]
	Zebrafish h*DNM2*-WT, S619L	Motor function impaired at 2dpf (higher impact mutant).	Fiber hypotrophy. Disorganized perinuclear material.	Abnormal triads and deficient ECC Swollen organelles Abnormal NMJ	[39,102]
	Zebrafish h*DNM2*-R522H	Motor function impaired Dose-dependent 24 dpf mortality.	Fiber size variability and increased central nuclei.	Abnormal NMJ	[104]
**Stable expression of *DNM2***	Mouse Tg MCK-rat *Dnm2* rat *Dnm2-*WT	Impaired motor function.	Fiber hypotrophy and centralized nuclei. Central accumulation of oxidative staining and radial strands.	Abnormal T-tubule	[99]
	Drosophila Tg h*DNM2*-WT R465W, S619L, A618T	Defects in the eclosion. Defective locomotor activity.	Fiber hypotrophy.	Abnormal T-tubule	[77]
	Zebrafish Tg(h*DNM2-EGFP*) WT,R465W,S619L	Impaired motor function (highest impact for S619L) from 2dpf.	Not specified.	Abnormal triad Abnormal NMJ	[103]
***DNM2*^R465W/+^** **dog**		Mildly progressive weakness.	Fiber size variability, central nuclei. Abnormal mitochondrial positioning: necklace fibers.	Not described yet	[105]

dpf: days post fertilization, E: embryonic day, P: postnatal day, ECC: excitation–contraction coupling, NMJ: neuromuscular junction, PI(3)P: Phosphatidylinositol 3-phosphate, Tg: transgenic.

**Table 4:** Main fish and mammalian models for BIN1-related ARCNM.

Genotype and Specie	Skeletal Muscle Phenotypes		Altered Pathways in Muscle	Ref (s)
	Lifespan and Motor Phenotypes	Muscle Histology		
***Bin1^−/−^*** mouse (*Bin1*ex3-6 deletion)	Perinatal death at P0.	Skeletal muscle not examined in detail.	Non investigated	[132]
***Bin1e*x20^−/−^** mouse (*Bin1*ex20 deletion)	Perinatal death at P0, feeding defect, no difference in body weight.	Centralized nuclei Central collapse of oxidative staining.	Abnormal triads	[84,131]
***Bin1e*x20^hsa−/−^** mouse (***Bin1e*x20^skm−/−^**, *Bin1*ex20 skeletal muscle deletion from E9)	Perinatal death at P0, feeding defect.	Not described.	Not described	[84,131]
***Bin1e*x20^mck−/−^** mouse (*Bin1ex20* skeletal muscle deletion from E17)	Normal lifespan, decreased body weight (from 4 months) and progressive moderate myopathy (from 8 weeks).	Fiber hypotrophy, normal nuclei position until 8 months. Central accumulation of oxidative staining.	Abnormal triads and defective ECC Defective autophagy	[135]
***Bin1e*x20^hsa(i)−/−^** mouse (***Bin1e*x20^skm(i)−/−^** deletion in adult skeletal muscle)	No impact on lifespan or body weight and normal motor function.	Muscle histology comparable to WT.	None	[131]
***Bin1*shRNA knock-down** in adult muscle	Not described.	Not described.	Abnormal triads and defective ECC	[134]
***Bin1e*x11^−/−^** mouse (splice switching from muscle-specific to ubiquitous isoform)	Normal lifespan and body weight and normal motor function.	Muscle histology comparable to WT. Slight, but significant, increased in mis-localized nuclei.	Satellite cell deficiency and defective muscle regeneration	[131]
***Bin1*ex11 transient skipping** by U7-ex11 AS in mouse	Reduced muscle force.	Histology comparable with WT.	Abnormal T-tubule orientation	[122]
***bin1*morphant** zebrafish	Defective motor function (from 17–26 hpf).	Mislocalized, rounded and grouped nuclei.	Abnormal triads and defective ECC	[136]
***BIN1*ex11 splice acceptor mutation** in dog (decreased BIN1 expression)	Highly progressive myopathy.	Fiber hypotrophy, internalized nuclei. Central accumulation of oxidative staining and some radial strands.	Abnormal triads Defective autophagy	[40]

hpf: hours post fertilization, dpf: days post fertilization, E: embryonic day, P: postnatal day, ECC: excitation–contraction coupling, NMJ: neuromuscular junction, skm: skeletal muscle, MCK: muscle creatine kinase, HSA: human skeletal actin.

**Table 5:** Main fish and mammalian models for RYR1-related ARCNM.

Genotype and Specie	Skeletal Muscle Phenotypes		Altered Pathways in Muscle	Ref (s)
	Lifespan and Motor Phenotypes	Muscle Histology		
***Ryr1*^TM/Indel^** mouse (compound heterozygous mutation)	Short lifespan (6-8 weeks), decreased body weight, progressive and severe myopathy.	Fiber hypotrophy, some centralized nuclei. No cores.	Defective ECC	[144]
***Ryr1*^Q1970fsX16+A4329D^** mouse (compound heterozygous mutation)	Normal lifespan, decreased body weight and moderate myopathy (from 3–4 months)/	Hypotrophy of type 2 fibers, nuclei position not described. Cores.	Abnormal triads and defective ECC Epigenetic dysregulation	[145]
**skr^m1^/skr^m1^** mouse (*Ryr1* null mutation, dyspedic mouse)	Perinatal death, respiratory failure.	Fiber hypotrophy.	Abnormal triads and defective ECC	[146,147]
***Ryr1-Rec*** mouse (*Ryr1* deletion in adult skeletal muscle)	Progressive myopathy, body weight reduction.	Fiber hypotrophy, normal nuclei position. Accumulation/depletion oxidative staining. “Dusty” cores.	Abnormal triads (multiple triads) and defective ECC Defective autophagy	[43]
***ryr1b^mi340^*** ***relatively relaxed* zebrafish mutant**	Decreased motor function (from 36 hpf) and lethality at 7–15 dpf.	Amorphous cores.	Abnormal triads and defective ECC Oxidative stress	[150,151]
***ryr1a;ryr1b*** **zebrafish double-mutant**	Complete paralysis and lethality at 7 dpf.	Not described.	Defective ECC	[152]

hpf: hours post fertilization, dpf: days post fertilization, E: embryonic day, P: postnatal day, ECC: excitation–contraction coupling, NMJ: neuromuscular junction, skm: skeletal muscle, MCK: muscle creatine kinase, has: human skeletal actin.

**Table 6:** List of therapeutic approaches and targets.

Approach	Purpose	CNM Form/Model	Therapeutic Effect Observed	Ref(s)	Status
**DNM2 reduction with ASO**	Normalization/reduction of DNM2	XLMTM/ *Mtm1*^−/y^ mouse	Prevention and reversion of CNM phenotypes: lifespan prolongation and rescue of body weight, improvement/rescue of muscle mass, histology, force, motor function and histology.	[236]	**Phase 1/2 clinical trial** initiated in 2020 in MTM1 and DNM2 patients (UNITE-CNM: NCT04033159)
		AD-CNM/*Dnm2* ^R465W/+^ mouse	Rescue of muscle mass and histology.	[191]	
		AD-CNM/*Dnm2* ^S619L/+^ mouse	Reversion force, motor function, fiber size and histology phenotypes.	[94]	
		AR-CNM/ *Bin1e*x20^mck−/−^ mouse	Improvement of force, fiber size and histology phenotypes.	[135]	
**DNM2 reduction with AAV-shRNA**	Normalization/reduction of DNM2	XLMTM/ *Mtm1*^−/y^ mouse	Improvement of muscle mass, force and rescue of histology.	[184]	**Preclinical studies**
		AD-CNM/*Dnm2* ^R465W/+^ mouse	Rescue of muscle mass and histology.	[191]	
	Specific reduction of *Dnm2*-R465W transcript	AD-CNM/*Dnm2* ^R465W/+^ mouse	Rescue of muscle mass, force and histology.	[237]	**Preclinical studies**
**Acetylcholine esterase inhibitor** (AChEI: Edrophonium, pyridostigmine)	NMJ transmission improvement	XLMTM/ *Mtm1*^R69C/y^ mouse	Improvement of motor function including exercise intolerance and fatigability.	[68]	FDA-approved drug. **Use in clinic** for myopathies as symptomatic treatment. Alleviated fatigability and improved strength in *MTM1* and *DNM2* patients [39,224]
		XLMTM/ *mtm1* morphant zebrafish	Fast improvement in motor function.	[224]	
		AD-CNM/hDNM2-S619L zebrafish (transient expression)	Rescue of the motor function.	[39]	
***MTM1* gene replacement** (AAV-MTM1)	MTM1 expression	XLMTM/ *Mtm1*^−/y^ mouse	Prevention and reversion of CNM phenotypes: lifespan prolongation, rescue of body weight and histology, improvement in muscle mass, force, and motor function.	[238,239]	**Phase 1/2 clinical trial** initiated in 2017 in MTM1 patients (ASPIRO: NCT03199469). First results showing striking muscular improvements. On hold due to fatal serious adverse events
		XLMTM/ XLMTM dog	Prolongation of survival, improve/rescue muscle mass, force, histology, and respiratory function	[238,240]	
**Myotubularin replacement** (3E10Fv-MTM1)	MTM1 re-expression	XLMTM/ *Mtm1*^−/y^ mouse	Intramuscular injection slightly improves muscle force. No amelioration of muscle histology.	[241]	**Preclinical studies** by Valerion Therapeutics
**MTMR2 gene therapy** (AAV-*MTMR2*)	MTMR2 expression	XLMTM/ *Mtm1*^−/y^ mouse	Lifespan prolongation, improvement in body weight, muscle mass, force and histology. Better rescue with short isoform of MTMR2 (MTMR2-S).	[242,243]	**Preclinical studies**
**BIN1 gene therapy** (AAV-*BIN1*)	BIN1 expression	XLMTM/ *Mtm1*^−/y^ mouse	Lifespan prolongation and rescue/improvement in muscle mass, force and histology.	[73]	**Preclinical studies**
**Allele-specific RNA silencing** (AAV-shRNA against *Dnm2*-R465W)	Reduction of mutated DNM2	AD-CNM/*Dnm2* ^R465W/+^ mouse	Rescued in muscle mass, specific force and histology.	[237]	**Preclinical studies**
**Exon skipping** (ASO to skip pseudo-exon)	Increase RyR1 protein level	AR-CNM/ Fetal primary muscle cells carrying *RyR1* patient mutation	Restore of calcium release from SR.	[244]	**Cell studies**
**Cell transplantation**	Muscle regeneration	XLMTM/ *Mtm1*^R69C/y^ mouse	Improvement in muscle strength and mass.	[245]	**Preclinical studies**
**Myostatin inhibition** (ActRIIB-mFC)	Muscle growth signaling pathway	XLMTM/ *Mtm1*^−/y^ mouse	Slight prolongation of lifespan, increase in muscle weight and transient slight improvement of muscle force.	[246]	**Preclinical studies**
		XLMTM/ *Mtm1*^R69C/y^ mouse	Increased gastrocnemius weight. No other improvements noted.	[74]	
**mTORC1 inhibition** (AZD8055)	Autophagy activation	XLMTM/ *Mtm1^gt^*^/*y*^	Restoration of muscle mass.	[61]	**Preclinical studies**
**PI3K inhibition** (Wortmannin)	Decreased PI(3)P levels	XLMTM/ *Mtm1*^−/y^ mouse	Lifespan prolongation and improvement of muscle histology. No improvement in body weight.	[63,70]	**Preclinical studies**
		XLMTM/ *mtm1* morphant zebrafish	Lifespan prolongation and improved motor function.	[63]	
**p38MAPK inhibition**	Drug repurposing, mechanism of action to be investigated	AR-CNM/ *ryr relatively relaxed* zebrafish	Not therapeutic improvement of motor function, although positive chemical-genetic interactions.	[247]	**Preclinical studies**
**Antioxidant N-acetylcysteine** (NAC)	Drug repurposing, decreased oxidative stress	AR-CNM/ *ryr relatively relaxed* zebrafish	Restore motor function and improve histology.	[151]	**Phase 1/2 clinical trial** initiated in 2015 and completed in 2018. Neither elevated oxidative stress nor exercise intolerance were rescued [248]
**Tamoxifen** (Estrogen receptor modulator)	Drug repurposing, pathways to be investigated.	XLMTM/ *Mtm1*^−/y^ mouse	Lifespan prolongation and delay of disease progression: improvement in muscle force, histology and motor function. No rescue of body weight.	[221,249]	**Phase 1/2 clinical trial** initiated at the end of 2020 in *MTM1* patients (TAM4MTM: NCT04915846)

## References

[1] Romero N.B. (2010). Centronuclear myopathies: A widening concept. Neuromuscul. Disord..

[2] Spiro A.J., Shy G.M., Gonatas N.K. (1966). Myotubular myopathy. Persistence of fetal muscle in an adolescent boy. Arch. Neurol..

[3] Laporte J., Hu L.J., Kretz C., Mandel J.L., Kioschis P., Coy J.F., Klauck S.M., Poustka A., Dahl N. (1996). A gene mutated in X-linked myotubular myopathy defines a new putative tyrosine phosphatase family conserved in yeast. Nat. Genet..

[4] Bitoun M., Maugenre S., Jeannet P.Y., Lacene E., Ferrer X., Laforet P., Martin J.J., Laporte J., Lochmuller H., Beggs A.H. (2005). Mutations in dynamin 2 cause dominant centronuclear myopathy. Nat. Genet..

[5] Bohm J., Biancalana V., Malfatti E., Dondaine N., Koch C., Vasli N., Kress W., Strittmatter M., Taratuto A.L., Gonorazky H. (2014). Adult-onset autosomal dominant centronuclear myopathy due to BIN1 mutations. Brain.

[6] Nicot A.S., Toussaint A., Tosch V., Kretz C., Wallgren-Pettersson C., Iwarsson E., Kingston H., Garnier J.M., Biancalana V., Oldfors A. (2007). Mutations in amphiphysin 2 (BIN1) disrupt interaction with dynamin 2 and cause autosomal recessive centronuclear myopathy. Nat. Genet..

[7] Bevilacqua J.A., Monnier N., Bitoun M., Eymard B., Ferreiro A., Monges S., Lubieniecki F., Taratuto A.L., Laquerriere A., Claeys K.G. (2011). Recessive RYR1 mutations cause unusual congenital myopathy with prominent nuclear internalization and large areas of myofibrillar disorganization. Neuropathol. Appl. Neurobiol..

[8] Wilmshurst J.M., Lillis S., Zhou H., Pillay K., Henderson H., Kress W., Muller C.R., Ndondo A., Cloke V., Cullup T. (2010). RYR1 mutations are a common cause of congenital myopathies with central nuclei. Ann. Neurol..

[9] Vandersmissen I., Biancalana V., Servais L., Dowling J.J., Vander Stichele G., Van Rooijen S., Thielemans L. (2018). An integrated modelling methodology for estimating the prevalence of centronuclear myopathy. Neuromuscul. Disord..

[10] Ceyhan-Birsoy O., Agrawal P.B., Hidalgo C., Schmitz-Abe K., DeChene E.T., Swanson L.C., Soemedi R., Vasli N., Iannaccone S.T., Shieh P.B. (2013). Recessive truncating titin gene, TTN, mutations presenting as centronuclear myopathy. Neurology.

[11] Agrawal P.B., Pierson C.R., Joshi M., Liu X., Ravenscroft G., Moghadaszadeh B., Talabere T., Viola M., Swanson L.C., Haliloglu G. (2014). SPEG Interacts with Myotubularin, and Its Deficiency Causes Centronuclear Myopathy with Dilated Cardiomyopathy. Am. J. Hum. Genet..

[12] Schartner V., Romero N.B., Donkervoort S., Treves S., Munot P., Pierson T.M., Dabaj I., Malfatti E., Zaharieva I.T., Zorzato F. (2017). Dihydropyridine receptor (DHPR, CACNA1S) congenital myopathy. Acta Neuropathol..

[13] Vasli N., Harris E., Karamchandani J., Bareke E., Majewski J., Romero N.B., Stojkovic T., Barresi R., Tasfaout H., Charlton R. (2017). Recessive mutations in the kinase ZAK cause a congenital myopathy with fibre type disproportion. Brain.

[14] Biancalana V., Caron O., Gallati S., Baas F., Kress W., Novelli G., D’Apice M.R., Lagier-Tourenne C., Buj-Bello A., Romero N.B. (2003). Characterisation of mutations in 77 patients with X-linked myotubular myopathy, including a family with a very mild phenotype. Hum. Genet..

[15] Buj-Bello A., Biancalana V., Moutou C., Laporte J., Mandel J.L. (1999). Identification of novel mutations in the MTM1 gene causing severe and mild forms of X-linked myotubular myopathy. Hum. Mutat..

[16] Herman G.E., Kopacz K., Zhao W., Mills P.L., Metzenberg A., Das S. (2002). Characterization of mutations in fifty North American patients with X- linked myotubular myopathy. Hum. Mutat..

[17] Laporte J., Biancalana V., Tanner S.M., Kress W., Schneider V., Wallgren-Pettersson C., Herger F., Buj-Bello A., Blondeau F., Liechti-Gallati S. (2000). MTM1 mutations in X-linked myotubular myopathy. Hum. Mutat..

[18] Tsai T.C., Horinouchi H., Noguchi S., Minami N., Murayama K., Hayashi Y.K., Nonaka I., Nishino I. (2005). Characterization of MTM1 mutations in 31 Japanese families with myotubular myopathy, including a patient carrying 240 kb deletion in Xq28 without male hypogenitalism. Neuromuscul. Disord..

[19] Laporte J., Kress W., Mandel J.L. (2001). Diagnosis of X-linked myotubular myopathy by detection of myotubularin. Ann. Neurol..

[20] Tosch V., Vasli N., Kretz C., Nicot A.S., Gasnier C., Dondaine N., Oriot D., Barth M., Puissant H., Romero N.B. (2010). Novel molecular diagnostic approaches for X-linked centronuclear (myotubular) myopathy reveal intronic mutations. Neuromuscul. Disord..

[21] Jungbluth H., Wallgren-Pettersson C., Laporte J. (2008). Centronuclear (myotubular) myopathy. Orphanet J. Rare Dis..

[22] Romero N.B., Bitoun M. (2011). Centronuclear myopathies. Semin. Pediatr. Neurol..

[23] Herman G.E., Finegold M., Zhao W., de Gouyon B., Metzenberg A. (1999). Medical complications in long-term survivors with X-linked myotubular myopathy. J. Pediatr..

[24] D’Amico A., Longo A., Fattori F., Tosi M., Bosco L., Testa M.B.C., Paglietti G., Cherchi C., Carlesi A., Mizzoni I. (2021). Hepatobiliary disease in XLMTM. A common comorbidity with potential impact on treatment strategies. Orphanet J. Rare Dis..

[25] Molera C., Sarishvili T., Nascimento A., Rtskhiladze I., Munoz Bartolo G., Fernandez Cebrian S., Valverde Fernandez J., Munoz Cabello B., Graham R.J., Miller W. (2021). Intrahepatic Cholestasis Is a Clinically Significant Feature Associated with Natural History of X-Linked Myotubular Myopathy (XLMTM): A Case Series and Biopsy Report. J. Neuromuscul. Dis..

[26] Biancalana V., Scheidecker S., Miguet M., Laquerriere A., Romero N.B., Stojkovic T., Abath Neto O., Mercier S., Voermans N., Tanner L. (2017). Affected female carriers of MTM1 mutations display a wide spectrum of clinical and pathological involvement: Delineating diagnostic clues. Acta Neuropathol..

[27] Cocanougher B.T., Flynn L., Yun P., Jain M., Waite M., Vasavada R., Wittenbach J.D., de Chastonay S., Chhibber S., Innes A.M. (2019). Adult MTM1-related myopathy carriers: Classification based on deep phenotyping. Neurology.

[28] Reumers S.F.I., Braun F., Spillane J.E., Bohm J., Pennings M., Schouten M., van der Kooi A.J., Foley A.R., Bonnemann C.G., Kamsteeg E.J. (2021). Spectrum of Clinical Features in X-Linked Myotubular Myopathy Carriers: An International Questionnaire Study. Neurology.

[29] Bachmann C., Jungbluth H., Muntoni F., Manzur A.Y., Zorzato F., Treves S. (2017). Cellular, biochemical and molecular changes in muscles from patients with X-linked myotubular myopathy due to MTM1 mutations. Hum. Mol. Genet..

[30] Bevilacqua J.A., Bitoun M., Biancalana V., Oldfors A., Stoltenburg G., Claeys K.G., Lacene E., Brochier G., Manere L., Laforet P. (2009). “Necklace” fibers, a new histological marker of late-onset MTM1-related centronuclear myopathy. Acta Neuropathol..

[31] Hnia K., Tronchere H., Tomczak K.K., Amoasii L., Schultz P., Beggs A.H., Payrastre B., Mandel J.L., Laporte J. (2011). Myotubularin controls desmin intermediate filament architecture and mitochondrial dynamics in human and mouse skeletal muscle. J. Clin. Investig..

[32] Shichiji M., Biancalana V., Fardeau M., Hogrel J.Y., Osawa M., Laporte J., Romero N.B. (2013). Extensive morphological and immunohistochemical characterization in myotubular myopathy. Brain Behav..

[33] Toussaint A., Cowling B.S., Hnia K., Mohr M., Oldfors A., Schwab Y., Yis U., Maisonobe T., Stojkovic T., Wallgren-Pettersson C. (2011). Defects in amphiphysin 2 (BIN1) and triads in several forms of centronuclear myopathies. Acta Neuropathol..

[34] Dowling J.J., Vreede A.P., Low S.E., Gibbs E.M., Kuwada J.Y., Bonnemann C.G., Feldman E.L. (2009). Loss of myotubularin function results in T-tubule disorganization in zebrafish and human myotubular myopathy. PLoS Genet..

[35] Ketel K., Krauss M., Nicot A.S., Puchkov D., Wieffer M., Muller R., Subramanian D., Schultz C., Laporte J., Haucke V. (2016). A phosphoinositide conversion mechanism for exit from endosomes. Nature.

[36] Ribeiro I., Yuan L., Tanentzapf G., Dowling J.J., Kiger A. (2011). Phosphoinositide regulation of integrin trafficking required for muscle attachment and maintenance. PLoS Genet..

[37] Bitoun M., Bevilacqua J.A., Eymard B., Prudhon B., Fardeau M., Guicheney P., Romero N.B. (2009). A new centronuclear myopathy phenotype due to a novel dynamin 2 mutation. Neurology.

[38] Bitoun M., Bevilacqua J.A., Prudhon B., Maugenre S., Taratuto A.L., Monges S., Lubieniecki F., Cances C., Uro-Coste E., Mayer M. (2007). Dynamin 2 mutations cause sporadic centronuclear myopathy with neonatal onset. Ann. Neurol..

[39] Gibbs E.M., Clarke N.F., Rose K., Oates E.C., Webster R., Feldman E.L., Dowling J.J. (2013). Neuromuscular junction abnormalities in DNM2-related centronuclear myopathy. J. Mol. Med..

[40] Bohm J., Vasli N., Maurer M., Cowling B., Shelton G.D., Kress W., Toussaint A., Prokic I., Schara U., Anderson T.J. (2013). Altered Splicing of the BIN1 Muscle-Specific Exon in Humans and Dogs with Highly Progressive Centronuclear Myopathy. PLoS Genet..

[41] D’Alessandro M., Hnia K., Gache V., Koch C., Gavriilidis C., Rodriguez D., Nicot A.S., Romero N.B., Schwab Y., Gomes E. (2015). Amphiphysin 2 Orchestrates Nucleus Positioning and Shape by Linking the Nuclear Envelope to the Actin and Microtubule Cytoskeleton. Dev. Cell.

[42] Abath Neto O., Moreno C.A.M., Malfatti E., Donkervoort S., Bohm J., Guimaraes J.B., Foley A.R., Mohassel P., Dastgir J., Bharucha-Goebel D.X. (2017). Common and variable clinical, histological, and imaging findings of recessive RYR1-related centronuclear myopathy patients. Neuromuscul. Disord..

[43] Pelletier L., Petiot A., Brocard J., Giannesini B., Giovannini D., Sanchez C., Travard L., Chivet M., Beaufils M., Kutchukian C. (2020). In vivo RyR1 reduction in muscle triggers a core-like myopathy. Acta Neuropathol. Commun..

[44] Rokach O., Sekulic-Jablanovic M., Voermans N., Wilmshurst J., Pillay K., Heytens L., Zhou H., Muntoni F., Gautel M., Nevo Y. (2015). Epigenetic changes as a common trigger of muscle weakness in congenital myopathies. Hum. Mol. Genet..

[45] De Craene J.O., Bertazzi D.L., Bar S., Friant S. (2017). Phosphoinositides, Major Actors in Membrane Trafficking and Lipid Signaling Pathways. Int. J. Mol. Sci..

[46] Vicinanza M., D’Angelo G., Di Campli A., De Matteis M.A. (2008). Function and dysfunction of the PI system in membrane trafficking. EMBO J..

[47] Blondeau F., Laporte J., Bodin S., Superti-Furga G., Payrastre B., Mandel J.L. (2000). Myotubularin, a phosphatase deficient in myotubular myopathy, acts on phosphatidylinositol 3-kinase and phosphatidylinositol 3-phosphate pathway. Hum. Mol. Genet..

[48] Taylor G.S., Maehama T., Dixon J.E. (2000). Inaugural article: Myotubularin, a protein tyrosine phosphatase mutated in myotubular myopathy, dephosphorylates the lipid second messenger, phosphatidylinositol 3-phosphate. Proc. Natl. Acad. Sci. USA.

[49] Tronchere H., Laporte J., Pendaries C., Chaussade C., Liaubet L., Pirola L., Mandel J.L., Payrastre B. (2004). Production of phosphatidylinositol 5-phosphate by the phosphoinositide 3-phosphatase myotubularin in mammalian cells. J. Biol. Chem..

[50] Laporte J., Blondeau F., Buj-Bello A., Tentler D., Kretz C., Dahl N., Mandel J.L. (1998). Characterization of the myotubularin dual specificity phosphatase gene family from yeast to human. Hum. Mol. Genet..

[51] Raess M.A., Friant S., Cowling B.S., Laporte J. (2017). WANTED—Dead or alive: Myotubularins, a large disease-associated protein family. Adv. Biol. Regul..

[52] Tsujita K., Itoh T., Ijuin T., Yamamoto A., Shisheva A., Laporte J., Takenawa T. (2004). Myotubularin regulates the function of the late endosome through the gram domain-phosphatidylinositol 3,5-bisphosphate interaction. J. Biol. Chem..

[53] Laporte J., Blondeau F., Gansmuller A., Lutz Y., Vonesch J.L., Mandel J.L. (2002). The PtdIns3P phosphatase myotubularin is a cytoplasmic protein that also localizes to Rac1-inducible plasma membrane ruffles. J. Cell Sci..

[54] Cui X., De Vivo I., Slany R., Miyamoto A., Firestein R., Cleary M.L. (1998). Association of SET domain and myotubularin-related proteins modulates growth control. Nat. Genet..

[55] Amburgey K., Tsuchiya E., de Chastonay S., Glueck M., Alverez R., Nguyen C.T., Rutkowski A., Hornyak J., Beggs A.H., Dowling J.J. (2017). A natural history study of X-linked myotubular myopathy. Neurology.

[56] Fattori F., Maggi L., Bruno C., Cassandrini D., Codemo V., Catteruccia M., Tasca G., Berardinelli A., Magri F., Pane M. (2015). Centronuclear myopathies: Genotype-phenotype correlation and frequency of defined genetic forms in an Italian cohort. J. Neurol..

[57] McEntagart M., Parsons G., Buj-Bello A., Biancalana V., Fenton I., Little M., Krawczak M., Thomas N., Herman G., Clarke A. (2002). Genotype-phenotype correlations in X-linked myotubular myopathy. Neuromuscul. Disord..

[58] Al-Qusairi L., Laporte J. (2011). T-tubule biogenesis and triad formation in skeletal muscle and implication in human diseases. Skelet. Muscle.

[59] Buj-Bello A., Laugel V., Messaddeq N., Zahreddine H., Laporte J., Pellissier J.F., Mandel J.L. (2002). The lipid phosphatase myotubularin is essential for skeletal muscle maintenance but not for myogenesis in mice. Proc. Natl. Acad. Sci. USA.

[60] Pierson C.R., Dulin-Smith A.N., Durban A.N., Marshall M.L., Marshall J.T., Snyder A.D., Naiyer N., Gladman J.T., Chandler D.S., Lawlor M.W. (2012). Modeling the human MTM1 p.R69C mutation in murine Mtm1 results in exon 4 skipping and a less severe myotubular myopathy phenotype. Hum. Mol. Genet..

[61] Fetalvero K.M., Yu Y., Goetschkes M., Liang G., Valdez R.A., Gould T., Triantafellow E., Bergling S., Loureiro J., Eash J. (2013). Defective autophagy and mTORC1 signaling in myotubularin null mice. Mol. Cell Biol..

[62] Chen X., Gao Y.Q., Zheng Y.Y., Wang W., Wang P., Liang J., Zhao W., Tao T., Sun J., Wei L. (2020). The intragenic microRNA miR199A1 in the dynamin 2 gene contributes to the pathology of X-linked centronuclear myopathy. J. Biol. Chem..

[63] Sabha N., Volpatti J.R., Gonorazky H., Reifler A., Davidson A.E., Li X., Eltayeb N.M., Dall’Armi C., Di Paolo G., Brooks S.V. (2016). PIK3C2B inhibition improves function and prolongs survival in myotubular myopathy animal models. J. Clin. Investig..

[64] Beggs A.H., Bohm J., Snead E., Kozlowski M., Maurer M., Minor K., Childers M.K., Taylor S.M., Hitte C., Mickelson J.R. (2010). MTM1 mutation associated with X-linked myotubular myopathy in Labrador Retrievers. Proc. Natl. Acad. Sci. USA.

[65] Shelton G.D., Rider B.E., Child G., Tzannes S., Guo L.T., Moghadaszadeh B., Troiano E.C., Haase B., Wade C.M., Beggs A.H. (2015). X-linked myotubular myopathy in Rottweiler dogs is caused by a missense mutation in Exon 11 of the MTM1 gene. Skelet. Muscle.

[66] Al-Qusairi L., Prokic I., Amoasii L., Kretz C., Messaddeq N., Mandel J.L., Laporte J. (2013). Lack of myotubularin (MTM1) leads to muscle hypotrophy through unbalanced regulation of the autophagy and ubiquitin-proteasome pathways. FASEB J..

[67] Al-Qusairi L., Weiss N., Toussaint A., Berbey C., Messaddeq N., Kretz C., Sanoudou D., Beggs A.H., Allard B., Mandel J.L. (2009). T-tubule disorganization and defective excitation-contraction coupling in muscle fibers lacking myotubularin lipid phosphatase. Proc. Natl. Acad. Sci. USA.

[68] Dowling J.J., Joubert R., Low S.E., Durban A.N., Messaddeq N., Li X., Dulin-Smith A.N., Snyder A.D., Marshall M.L., Marshall J.T. (2012). Myotubular myopathy and the neuromuscular junction: A novel therapeutic approach from mouse models. Dis. Model. Mech..

[69] Gavriilidis C., Laredj L., Solinhac R., Messaddeq N., Viaud J., Laporte J., Sumara I., Hnia K. (2018). The MTM1-UBQLN2-HSP complex mediates degradation of misfolded intermediate filaments in skeletal muscle. Nat. Cell Biol..

[70] Kutchukian C., Lo Scrudato M., Tourneur Y., Poulard K., Vignaud A., Berthier C., Allard B., Lawlor M.W., Buj-Bello A., Jacquemond V. (2016). Phosphatidylinositol 3-kinase inhibition restores Ca^2+^ release defects and prolongs survival in myotubularin-deficient mice. Proc. Natl. Acad. Sci. USA.

[71] Kutchukian C., Szentesi P., Allard B., Buj-Bello A., Csernoch L., Jacquemond V. (2019). Ca(2+)-induced sarcoplasmic reticulum Ca(2+) release in myotubularin-deficient muscle fibers. Cell Calcium.

[72] Lawlor M.W., Alexander M.S., Viola M.G., Meng H., Joubert R., Gupta V., Motohashi N., Manfready R.A., Hsu C.P., Huang P. (2012). Myotubularin-deficient myoblasts display increased apoptosis, delayed proliferation, and poor cell engraftment. Am. J. Pathol..

[73] Lionello V.M., Nicot A.S., Sartori M., Kretz C., Kessler P., Buono S., Djerroud S., Messaddeq N., Koebel P., Prokic I. (2019). Amphiphysin 2 modulation rescues myotubular myopathy and prevents focal adhesion defects in mice. Sci. Transl. Med..

[74] Lawlor M.W., Viola M.G., Meng H., Edelstein R.V., Liu F., Yan K., Luna E.J., Lerch-Gaggl A., Hoffmann R.G., Pierson C.R. (2014). Differential muscle hypertrophy is associated with satellite cell numbers and Akt pathway activation following activin type IIB receptor inhibition in Mtm1 p.R69C mice. Am. J. Pathol..

[75] Ross J.A., Tasfaout H., Levy Y., Morgan J., Cowling B.S., Laporte J., Zanoteli E., Romero N.B., Lowe D.A., Jungbluth H. (2020). rAAV-related therapy fully rescues myonuclear and myofilament function in X-linked myotubular myopathy. Acta Neuropathol. Commun..

[76] Bohm J., Biancalana V., Dechene E.T., Bitoun M., Pierson C.R., Schaefer E., Karasoy H., Dempsey M.A., Klein F., Dondaine N. (2012). Mutation spectrum in the large GTPase dynamin 2, and genotype-phenotype correlation in autosomal dominant centronuclear myopathy. Hum. Mutat..

[77] Chin Y.H., Lee A., Kan H.W., Laiman J., Chuang M.C., Hsieh S.T., Liu Y.W. (2015). Dynamin-2 mutations associated with centronuclear myopathy are hypermorphic and lead to T-tubule fragmentation. Hum. Mol. Genet..

[78] Kenniston J.A., Lemmon M.A. (2010). Dynamin GTPase regulation is altered by PH domain mutations found in centronuclear myopathy patients. EMBO J..

[79] Wang L., Barylko B., Byers C., Ross J.A., Jameson D.M., Albanesi J.P. (2010). Dynamin 2 mutants linked to centronuclear myopathies form abnormally stable polymers. J. Biol. Chem..

[80] Susman R.D., Quijano-Roy S., Yang N., Webster R., Clarke N.F., Dowling J., Kennerson M., Nicholson G., Biancalana V., Ilkovski B. (2010). Expanding the clinical, pathological and MRI phenotype of DNM2-related centronuclear myopathy. Neuromuscul. Disord..

[81] Echaniz-Laguna A., Nicot A.S., Carre S., Franques J., Tranchant C., Dondaine N., Biancalana V., Mandel J.L., Laporte J. (2007). Subtle central and peripheral nervous system abnormalities in a family with centronuclear myopathy and a novel dynamin 2 gene mutation. Neuromuscul. Disord..

[82] Fischer D., Herasse M., Bitoun M., Barragan-Campos H.M., Chiras J., Laforet P., Fardeau M., Eymard B., Guicheney P., Romero N.B. (2006). Characterization of the muscle involvement in dynamin 2-related centronuclear myopathy. Brain.

[83] Ferguson S.M., De Camilli P. (2012). Dynamin, a membrane-remodelling GTPase. Nat. Rev. Mol. Cell Biol..

[84] Cowling B.S., Prokic I., Tasfaout H., Rabai A., Humbert F., Rinaldi B., Nicot A.S., Kretz C., Friant S., Roux A. (2017). Amphiphysin (BIN1) negatively regulates dynamin 2 for normal muscle maturation. J. Clin. Investig..

[85] McNiven M.A., Cao H., Pitts K.R., Yoon Y. (2000). The dynamin family of mechanoenzymes: Pinching in new places. Trends Biochem. Sci..

[86] Warnock D.E., Baba T., Schmid S.L. (1997). Ubiquitously expressed dynamin-II has a higher intrinsic GTPase activity and a greater propensity for self-assembly than neuronal dynamin-I. Mol. Biol. Cell.

[87] Gu C., Yaddanapudi S., Weins A., Osborn T., Reiser J., Pollak M., Hartwig J., Sever S. (2010). Direct dynamin-actin interactions regulate the actin cytoskeleton. EMBO J..

[88] Klein D.E., Lee A., Frank D.W., Marks M.S., Lemmon M.A. (1998). The pleckstrin homology domains of dynamin isoforms require oligomerization for high affinity phosphoinositide binding. J. Biol. Chem..

[89] Reubold T.F., Faelber K., Plattner N., Posor Y., Ketel K., Curth U., Schlegel J., Anand R., Manstein D.J., Noé F. (2015). Crystal structure of the dynamin tetramer. Nature.

[90] Antonny B., Burd C., De Camilli P., Chen E., Daumke O., Faelber K., Ford M., Frolov V.A., Frost A., Hinshaw J.E. (2016). Membrane fission by dynamin: What we know and what we need to know. EMBO J..

[91] James N.G., Digman M.A., Ross J.A., Barylko B., Wang L., Li J., Chen Y., Mueller J.D., Gratton E., Albanesi J.P. (2014). A mutation associated with centronuclear myopathy enhances the size and stability of dynamin 2 complexes in cells. Biochim. Biophys. Acta.

[92] Srinivasan S., Dharmarajan V., Reed D.K., Griffin P.R., Schmid S.L. (2016). Identification and function of conformational dynamics in the multidomain GTPase dynamin. EMBO J..

[93] Durieux A.C., Vignaud A., Prudhon B., Viou M.T., Beuvin M., Vassilopoulos S., Fraysse B., Ferry A., Laine J., Romero N.B. (2010). A centronuclear myopathy-dynamin 2 mutation impairs skeletal muscle structure and function in mice. Hum. Mol. Genet..

[94] Massana Munoz X., Kretz C., Silva-Rojas R., Ochala J., Menuet A., Romero N.B., Cowling B.S., Laporte J. (2020). Physiological impact and disease reversion for the severe form of centronuclear myopathy linked to dynamin. JCI Insight.

[95] Tinelli E., Pereira J.A., Suter U. (2013). Muscle-specific function of the centronuclear myopathy and Charcot-Marie-Tooth neuropathy-associated dynamin 2 is required for proper lipid metabolism, mitochondria, muscle fibers, neuromuscular junctions and peripheral nerves. Hum. Mol. Genet..

[96] Cowling B.S., Chevremont T., Prokic I., Kretz C., Ferry A., Coirault C., Koutsopoulos O., Laugel V., Romero N.B., Laporte J. (2014). Reducing dynamin 2 expression rescues X-linked centronuclear myopathy. J. Clin. Investig..

[97] Cowling B.S., Toussaint A., Amoasii L., Koebel P., Ferry A., Davignon L., Nishino I., Mandel J.L., Laporte J. (2011). Increased expression of wild-type or a centronuclear myopathy mutant of dynamin 2 in skeletal muscle of adult mice leads to structural defects and muscle weakness. Am. J. Pathol..

[98] Massana Munoz X., Buono S., Koebel P., Laporte J., Cowling B.S. (2019). Different in vivo impacts of dynamin 2 mutations implicated in Charcot-Marie-Tooth neuropathy or centronuclear myopathy. Hum. Mol. Genet..

[99] Liu N., Bezprozvannaya S., Shelton J.M., Frisard M.I., Hulver M.W., McMillan R.P., Wu Y., Voelker K.A., Grange R.W., Richardson J.A. (2011). Mice lacking microRNA 133a develop dynamin 2-dependent centronuclear myopathy. J. Clin. Investig..

[100] Suzuki D.T., Grigliatti T., Williamson R. (1971). Temperature-sensitive mutations in Drosophila melanogaster. VII. A mutation (para-ts) causing reversible adult paralysis. Proc. Natl. Acad. Sci. USA.

[101] van der Bliek A.M., Meyerowitz E.M. (1991). Dynamin-like protein encoded by the Drosophila shibire gene associated with vesicular traffic. Nature.

[102] Gibbs E.M., Davidson A.E., Telfer W.R., Feldman E.L., Dowling J.J. (2014). The myopathy-causing mutation DNM2-S619L leads to defective tubulation in vitro and in developing zebrafish. Dis. Model. Mech..

[103] Zhao M., Smith L., Volpatti J., Fabian L., Dowling J.J. (2019). Insights into wild type dynamin 2 and the consequences of DNM2 mutations from transgenic zebrafish. Hum. Mol. Genet..

[104] Bragato C., Gaudenzi G., Blasevich F., Pavesi G., Maggi L., Giunta M., Cotelli F., Mora M. (2016). Zebrafish as a Model to Investigate Dynamin 2-Related Diseases. Sci. Rep..

[105] Böhm J., Barthélémy I., Blot S., Tiret L., Laporte J. (2020). A dog model for centronuclear myopathy carrying the most common DNM2 mutation. Neuromuscul. Disord..

[106] Almeida C.F., Bitoun M., Vainzof M. (2021). Satellite cells deficiency and defective regeneration in dynamin 2-related centronuclear myopathy. FASEB J..

[107] Durieux A.C., Vassilopoulos S., Laine J., Fraysse B., Brinas L., Prudhon B., Castells J., Freyssenet D., Bonne G., Guicheney P. (2012). A centronuclear myopathy--dynamin 2 mutation impairs autophagy in mice. Traffic.

[108] Fongy A., Falcone S., Laine J., Prudhon B., Martins-Bach A., Bitoun M. (2019). Nuclear defects in skeletal muscle from a Dynamin 2-linked centronuclear myopathy mouse model. Sci. Rep..

[109] Franck A., Laine J., Moulay G., Lemerle E., Trichet M., Gentil C., Benkhelifa-Ziyyat S., Lacene E., Bui M.T., Brochier G. (2019). Clathrin plaques and associated actin anchor intermediate filaments in skeletal muscle. Mol. Biol. Cell.

[110] Fraysse B., Guicheney P., Bitoun M. (2016). Calcium homeostasis alterations in a mouse model of the Dynamin 2-related centronuclear myopathy. Biol. Open.

[111] Gonzalez-Jamett A.M., Baez-Matus X., Olivares M.J., Hinostroza F., Guerra-Fernandez M.J., Vasquez-Navarrete J., Bui M.T., Guicheney P., Romero N.B., Bevilacqua J.A. (2017). Dynamin-2 mutations linked to Centronuclear Myopathy impair actin-dependent trafficking in muscle cells. Sci. Rep..

[112] Kutchukian C., Szentesi P., Allard B., Trochet D., Beuvin M., Berthier C., Tourneur Y., Guicheney P., Csernoch L., Bitoun M. (2017). Impaired excitation-contraction coupling in muscle fibres from the dynamin2(R465W) mouse model of centronuclear myopathy. J. Physiol..

[113] Puri C., Manni M.M., Vicinanza M., Hilcenko C., Zhu Y., Runwal G., Stamatakou E., Menzies F.M., Mamchaoui K., Bitoun M. (2020). A DNM2 Centronuclear Myopathy Mutation Reveals a Link between Recycling Endosome Scission and Autophagy. Dev. Cell.

[114] Rabai A., Reisser L., Reina-San-Martin B., Mamchaoui K., Cowling B.S., Nicot A.S., Laporte J. (2019). Allele-Specific CRISPR/Cas9 Correction of a Heterozygous DNM2 Mutation Rescues Centronuclear Myopathy Cell Phenotypes. Mol. Ther. Nucleic Acids.

[115] Bohm J., Yis U., Ortac R., Cakmakci H., Kurul S.H., Dirik E., Laporte J. (2010). Case report of intrafamilial variability in autosomal recessive centronuclear myopathy associated to a novel BIN1 stop mutation. Orphanet J. Rare Dis..

[116] Cabrera-Serrano M., Mavillard F., Biancalana V., Rivas E., Morar B., Hernandez-Lain A., Olive M., Muelas N., Khan E., Carvajal A. (2018). A Roma founder BIN1 mutation causes a novel phenotype of centronuclear myopathy with rigid spine. Neurology.

[117] Claeys K.G., Maisonobe T., Bohm J., Laporte J., Hezode M., Romero N.B., Brochier G., Bitoun M., Carlier R.Y., Stojkovic T. (2010). Phenotype of a patient with recessive centronuclear myopathy and a novel BIN1 mutation. Neurology.

[118] Prokic I., Cowling B.S., Laporte J. (2014). Amphiphysin 2 (BIN1) in physiology and diseases. J. Mol. Med..

[119] Frost A., Unger V.M., De Camilli P. (2009). The BAR domain superfamily: Membrane-molding macromolecules. Cell.

[120] Peter B.J., Kent H.M., Mills I.G., Vallis Y., Butler P.J., Evans P.R., McMahon H.T. (2004). BAR domains as sensors of membrane curvature: The amphiphysin BAR structure. Science.

[121] Wechsler-Reya R., Sakamuro D., Zhang J., Duhadaway J., Prendergast G.C. (1997). Structural analysis of the human BIN1 gene. Evidence for tissue-specific transcriptional regulation and alternate RNA splicing. J. Biol. Chem..

[122] Fugier C., Klein A.F., Hammer C., Vassilopoulos S., Ivarsson Y., Toussaint A., Tosch V., Vignaud A., Ferry A., Messaddeq N. (2011). Misregulated alternative splicing of BIN1 is associated with T tubule alterations and muscle weakness in myotonic dystrophy. Nat. Med..

[123] Lee E., Marcucci M., Daniell L., Pypaert M., Weisz O.A., Ochoa G.C., Farsad K., Wenk M.R., De Camilli P. (2002). Amphiphysin 2 (Bin1) and T-tubule biogenesis in muscle. Science.

[124] Kojima C., Hashimoto A., Yabuta I., Hirose M., Hashimoto S., Kanaho Y., Sumimoto H., Ikegami T., Sabe H. (2004). Regulation of Bin1 SH3 domain binding by phosphoinositides. Embo J..

[125] Butler M.H., David C., Ochoa G.C., Freyberg Z., Daniell L., Grabs D., Cremona O., De Camilli P. (1997). Amphiphysin II (SH3P9; BIN1), a member of the amphiphysin/Rvs family, is concentrated in the cortical cytomatrix of axon initial segments and nodes of Ranvier in brain and around T tubules in skeletal muscle. J. Cell Biol..

[126] Ramjaun A.R., Micheva K.D., Bouchelet I., McPherson P.S. (1997). Identification and characterization of a nerve terminal-enriched amphiphysin isoform. J. Biol. Chem..

[127] Sakamuro D., Elliott K.J., Wechsler-Reya R., Prendergast G.C. (1996). BIN1 is a novel MYC-interacting protein with features of a tumour suppressor. Nat. Genet..

[128] Yu H., Chen J.K., Feng S., Dalgarno D.C., Brauer A.W., Schreiber S.L. (1994). Structural basis for the binding of proline-rich peptides to SH3 domains. Cell.

[129] Falcone S., Roman W., Hnia K., Gache V., Didier N., Laine J., Aurade F., Marty I., Nishino I., Charlet-Berguerand N. (2014). N-WASP is required for Amphiphysin-2/BIN1-dependent nuclear positioning and triad organization in skeletal muscle and is involved in the pathophysiology of centronuclear myopathy. EMBO Mol. Med..

[130] Royer B., Hnia K., Gavriilidis C., Tronchere H., Tosch V., Laporte J. (2013). The myotubularin-amphiphysin 2 complex in membrane tubulation and centronuclear myopathies. EMBO Rep..

[131] Prokic I., Cowling B.S., Kutchukian C., Kretz C., Tasfaout H., Gache V., Hergueux J., Wendling O., Ferry A., Toussaint A. (2020). Differential physiological role of BIN1 isoforms in skeletal muscle development, function and regeneration. Dis. Model. Mech..

[132] Muller A.J., Baker J.F., DuHadaway J.B., Ge K., Farmer G., Donover P.S., Meade R., Reid C., Grzanna R., Roach A.H. (2003). Targeted disruption of the murine Bin1/Amphiphysin II gene does not disable endocytosis but results in embryonic cardiomyopathy with aberrant myofibril formation. Mol. Cell Biol..

[133] Laury-Kleintop L.D., Mulgrew J.R., Heletz I., Nedelcoviciu R.A., Chang M.Y., Harris D.M., Koch W.J., Schneider M.D., Muller A.J., Prendergast G.C. (2015). Cardiac-specific disruption of Bin1 in mice enables a model of stress- and age-associated dilated cardiomyopathy. J. Cell Biochem..

[134] Tjondrokoesoemo A., Park K.H., Ferrante C., Komazaki S., Lesniak S., Brotto M., Ko J.K., Zhou J., Weisleder N., Ma J. (2011). Disrupted membrane structure and intracellular Ca(2)(+) signaling in adult skeletal muscle with acute knockdown of Bin1. PLoS ONE.

[135] Silva-Rojas R., Nattarayan V., Jaque-Fernandez F., Gomez-Oca R., Menuet A., Reiss D., Goret M., Messaddeq N., Lionello V.M., Kretz C. (2021). Mice with muscle-specific deletion of Bin1 recapitulate centronuclear myopathy and acute downregulation of dynamin 2 improves their phenotypes. Mol. Ther..

[136] Smith L.L., Gupta V.A., Beggs A.H. (2014). Bridging integrator 1 (Bin1) deficiency in zebrafish results in centronuclear myopathy. Hum. Mol. Genet..

[137] Davies S.E., Davies D.R., Richards R.B., Bruce W.J. (2008). Inherited myopathy in a Great Dane. Aust. Vet. J..

[138] Lujan Feliu-Pascual A., Shelton G.D., Targett M.P., Long S.N., Comerford E.J., McMillan C., Davies D., Rusbridge C., Mellor D., Chang K.C. (2006). Inherited myopathy of great Danes. J. Small Anim. Pract..

[139] McMillan C.J., Taylor S.M., Shelton G.D. (2006). Inherited myopathy in a young Great Dane. Can. Vet. J..

[140] Kushnir A., Wajsberg B., Marks A.R. (2018). Ryanodine receptor dysfunction in human disorders. Biochim. Biophys. Acta Mol. Cell Res..

[141] des Georges A., Clarke O.B., Zalk R., Yuan Q., Condon K.J., Grassucci R.A., Hendrickson W.A., Marks A.R., Frank J. (2016). Structural Basis for Gating and Activation of RyR1. Cell.

[142] Van Petegem F. (2012). Ryanodine receptors: Structure and function. J. Biol. Chem..

[143] Lawal T.A., Wires E.S., Terry N.L., Dowling J.J., Todd J.J. (2020). Preclinical model systems of ryanodine receptor 1-related myopathies and malignant hyperthermia: A comprehensive scoping review of works published 1990-2019. Orphanet J. Rare Dis..

[144] Brennan S., Garcia-Castaneda M., Michelucci A., Sabha N., Malik S., Groom L., Wei LaPierre L., Dowling J.J., Dirksen R.T. (2019). Mouse model of severe recessive RYR1-related myopathy. Hum. Mol. Genet..

[145] Elbaz M., Ruiz A., Bachmann C., Eckhardt J., Pelczar P., Venturi E., Lindsay C., Wilson A.D., Alhussni A., Humberstone T. (2019). Quantitative RyR1 reduction and loss of calcium sensitivity of RyR1Q1970fsX16+A4329D cause cores and loss of muscle strength. Hum. Mol. Genet..

[146] Takeshima H., Iino M., Takekura H., Nishi M., Kuno J., Minowa O., Takano H., Noda T. (1994). Excitation-contraction uncoupling and muscular degeneration in mice lacking functional skeletal muscle ryanodine-receptor gene. Nature.

[147] Takekura H., Nishi M., Noda T., Takeshima H., Franzini-Armstrong C. (1995). Abnormal junctions between surface membrane and sarcoplasmic reticulum in skeletal muscle with a mutation targeted to the ryanodine receptor. Proc. Natl. Acad. Sci. USA.

[148] Garibaldi M., Rendu J., Brocard J., Lacene E., Faure J., Brochier G., Beuvin M., Labasse C., Madelaine A., Malfatti E. (2019). ‘Dusty core disease’ (DuCD): Expanding morphological spectrum of RYR1 recessive myopathies. Acta Neuropathol. Commun..

[149] Cacheux M., Blum A., Sebastien M., Wozny A.S., Brocard J., Mamchaoui K., Mouly V., Roux-Buisson N., Rendu J., Monnier N. (2015). Functional Characterization of a Central Core Disease RyR1 Mutation (p.Y4864H) Associated with Quantitative Defect in RyR1 Protein. J. Neuromuscul. Dis..

[150] Hirata H., Watanabe T., Hatakeyama J., Sprague S.M., Saint-Amant L., Nagashima A., Cui W.W., Zhou W., Kuwada J.Y. (2007). Zebrafish relatively relaxed mutants have a ryanodine receptor defect, show slow swimming and provide a model of multi-minicore disease. Development.

[151] Dowling J.J., Arbogast S., Hur J., Nelson D.D., McEvoy A., Waugh T., Marty I., Lunardi J., Brooks S.V., Kuwada J.Y. (2012). Oxidative stress and successful antioxidant treatment in models of RYR1-related myopathy. Brain.

[152] Chagovetz A.A., Klatt Shaw D., Ritchie E., Hoshijima K., Grunwald D.J. (2019). Interactions among ryanodine receptor isotypes contribute to muscle fiber type development and function. Dis. Model. Mech..

[153] Djeddi S., Reiss D., Menuet A., Freismuth S., de Carvalho Neves J., Djerroud S., Massana-Munoz X., Sosson A.S., Kretz C., Raffelsberger W. (2021). Multi-omics comparisons of different forms of centronuclear myopathies and the effects of several therapeutic strategies. Mol. Ther..

[154] Dupont J.B., Guo J., Renaud-Gabardos E., Poulard K., Latournerie V., Lawlor M.W., Grange R.W., Gray J.T., Buj-Bello A., Childers M.K. (2020). AAV-Mediated Gene Transfer Restores a Normal Muscle Transcriptome in a Canine Model of X-Linked Myotubular Myopathy. Mol. Ther..

[155] Razzaq A., Robinson I.M., McMahon H.T., Skepper J.N., Su Y., Zelhof A.C., Jackson A.P., Gay N.J., O’Kane C.J. (2001). Amphiphysin is necessary for organization of the excitation-contraction coupling machinery of muscles, but not for synaptic vesicle endocytosis in Drosophila. Genes Dev..

[156] Takekura H., Flucher B.E., Franzini-Armstrong C. (2001). Sequential docking, molecular differentiation, and positioning of T-Tubule/SR junctions in developing mouse skeletal muscle. Dev. Biol..

[157] Sarnat H.B. (1990). Myotubular myopathy: Arrest of morphogenesis of myofibres associated with persistence of fetal vimentin and desmin. Four cases compared with fetal and neonatal muscle. Can. J. Neurol. Sci..

[158] Amoasii L., Hnia K., Chicanne G., Brech A., Cowling B.S., Muller M.M., Schwab Y., Koebel P., Ferry A., Payrastre B. (2013). Myotubularin and PtdIns3P remodel the sarcoplasmic reticulum in muscle in vivo. J. Cell Sci..

[159] Dirksen R.T., Avila G. (2002). Altered ryanodine receptor function in central core disease: Leaky or uncoupled Ca(2+) release channels?. Trends Cardiovasc. Med..

[160] Klatt Shaw D., Gunther D., Jurynec M.J., Chagovetz A.A., Ritchie E., Grunwald D.J. (2018). Intracellular Calcium Mobilization Is Required for Sonic Hedgehog Signaling. Dev. Cell.

[161] Fujise K., Okubo M., Abe T., Yamada H., Nishino I., Noguchi S., Takei K., Takeda T. (2021). Mutant BIN1-Dynamin 2 complexes dysregulate membrane remodeling in the pathogenesis of centronuclear myopathy. J. Biol. Chem..

[162] Di Biase V., Tuluc P., Campiglio M., Obermair G.J., Heine M., Flucher B.E. (2011). Surface traffic of dendritic CaV1.2 calcium channels in hippocampal neurons. J. Neurosci..

[163] Yang T., Xu X., Kernan T., Wu V., Colecraft H.M. (2010). Rem, a member of the RGK GTPases, inhibits recombinant CaV1.2 channels using multiple mechanisms that require distinct conformations of the GTPase. J. Physiol..

[164] Shen J., Yu W.M., Brotto M., Scherman J.A., Guo C., Stoddard C., Nosek T.M., Valdivia H.H., Qu C.K. (2009). Deficiency of MIP/MTMR14 phosphatase induces a muscle disorder by disrupting Ca(2+) homeostasis. Nat. Cell Biol..

[165] Tomasevic N., Jia Z., Russell A., Fujii T., Hartman J.J., Clancy S., Wang M., Beraud C., Wood K.W., Sakowicz R. (2007). Differential regulation of WASP and N-WASP by Cdc42, Rac1, Nck, and PI(4,5)P2. Biochemistry.

[166] Neukomm L.J., Nicot A.S., Kinchen J.M., Almendinger J., Pinto S.M., Zeng S., Doukoumetzidis K., Tronchere H., Payrastre B., Laporte J.F. (2011). The phosphoinositide phosphatase MTM-1 regulates apoptotic cell corpse clearance through CED-5-CED-12 in C. elegans. Development.

[167] Kessels M.M., Engqvist-Goldstein A.E., Drubin D.G., Qualmann B. (2001). Mammalian Abp1, a signal-responsive F-actin-binding protein, links the actin cytoskeleton to endocytosis via the GTPase dynamin. J. Cell Biol..

[168] Mooren O.L., Kotova T.I., Moore A.J., Schafer D.A. (2009). Dynamin2 GTPase and cortactin remodel actin filaments. J. Biol. Chem..

[169] Danowski B.A., Imanaka-Yoshida K., Sanger J.M., Sanger J.W. (1992). Costameres are sites of force transmission to the substratum in adult rat cardiomyocytes. J. Cell Biol..

[170] Ervasti J.M. (2003). Costameres: The Achilles’ heel of Herculean muscle. J. Biol. Chem..

[171] Vassilopoulos S., Gentil C., Laine J., Buclez P.O., Franck A., Ferry A., Precigout G., Roth R., Heuser J.E., Brodsky F.M. (2014). Actin scaffolding by clathrin heavy chain is required for skeletal muscle sarcomere organization. J. Cell Biol..

[172] Brinas L., Vassilopoulos S., Bonne G., Guicheney P., Bitoun M. (2013). Role of dynamin 2 in the disassembly of focal adhesions. J. Mol. Med..

[173] Ezratty E.J., Partridge M.A., Gundersen G.G. (2005). Microtubule-induced focal adhesion disassembly is mediated by dynamin and focal adhesion kinase. Nat. Cell Biol..

[174] Drager N.M., Nachman E., Winterhoff M., Bruhmann S., Shah P., Katsinelos T., Boulant S., Teleman A.A., Faix J., Jahn T.R. (2017). Bin1 directly remodels actin dynamics through its BAR domain. EMBO Rep..

[175] Aspenstrom P. (2014). BAR domain proteins regulate Rho GTPase signaling. Small GTPases.

[176] Cadot B., Gache V., Gomes E.R. (2015). Moving and positioning the nucleus in skeletal muscle—One step at a time. Nucleus.

[177] Shpetner H.S., Vallee R.B. (1989). Identification of dynamin, a novel mechanochemical enzyme that mediates interactions between microtubules. Cell.

[178] Shpetner H.S., Vallee R.B. (1992). Dynamin is a GTPase stimulated to high levels of activity by microtubules. Nature.

[179] Tanabe K., Takei K. (2009). Dynamic instability of microtubules requires dynamin 2 and is impaired in a Charcot-Marie-Tooth mutant. J. Cell Biol..

[180] Koutsopoulos O.S., Koch C., Tosch V., Bohm J., North K.N., Laporte J. (2011). Mild functional differences of dynamin 2 mutations associated to centronuclear myopathy and charcot-marie-tooth peripheral neuropathy. PLoS ONE.

[181] Agnetti G., Herrmann H., Cohen S. (2021). New roles for desmin in the maintenance of muscle homeostasis. FEBS J..

[182] Roman W., Martins J.P., Carvalho F.A., Voituriez R., Abella J.V.G., Santos N.C., Cadot B., Way M., Gomes E.R. (2017). Myofibril contraction and crosslinking drive nuclear movement to the periphery of skeletal muscle. Nat. Cell Biol..

[183] Amoasii L., Bertazzi D.L., Tronchere H., Hnia K., Chicanne G., Rinaldi B., Cowling B.S., Ferry A., Klaholz B., Payrastre B. (2012). Phosphatase-dead myotubularin ameliorates X-linked centronuclear myopathy phenotypes in mice. PLoS Genet..

[184] Tasfaout H., Lionello V.M., Kretz C., Koebel P., Messaddeq N., Bitz D., Laporte J., Cowling B.S. (2018). Single Intramuscular Injection of AAV-shRNA Reduces DNM2 and Prevents Myotubular Myopathy in Mice. Mol. Ther..

[185] Ralston E., Lu Z., Biscocho N., Soumaka E., Mavroidis M., Prats C., Lomo T., Capetanaki Y., Ploug T. (2006). Blood vessels and desmin control the positioning of nuclei in skeletal muscle fibers. J. Cell Physiol..

[186] Shah S.B., Davis J., Weisleder N., Kostavassili I., McCulloch A.D., Ralston E., Capetanaki Y., Lieber R.L. (2004). Structural and functional roles of desmin in mouse skeletal muscle during passive deformation. Biophys. J..

[187] Pierson C.R., Agrawal P.B., Blasko J., Beggs A.H. (2007). Myofiber size correlates with MTM1 mutation type and outcome in X-linked myotubular myopathy. Neuromuscul. Disord..

[188] Lee J.E., Westrate L.M., Wu H., Page C., Voeltz G.K. (2016). Multiple dynamin family members collaborate to drive mitochondrial division. Nature.

[189] Fonseca T.B., Sanchez-Guerrero A., Milosevic I., Raimundo N. (2019). Mitochondrial fission requires DRP1 but not dynamins. Nature.

[190] Kamerkar S.C., Kraus F., Sharpe A.J., Pucadyil T.J., Ryan M.T. (2018). Dynamin-related protein 1 has membrane constricting and severing abilities sufficient for mitochondrial and peroxisomal fission. Nat. Commun..

[191] Buono S., Ross J.A., Tasfaout H., Levy Y., Kretz C., Tayefeh L., Matson J., Guo S., Kessler P., Monia B.P. (2018). Reducing dynamin 2 (DNM2) rescues DNM2-related dominant centronuclear myopathy. Proc. Natl. Acad. Sci. USA.

[192] Milner D.J., Mavroidis M., Weisleder N., Capetanaki Y. (2000). Desmin cytoskeleton linked to muscle mitochondrial distribution and respiratory function. J. Cell Biol..

[193] Kosaka T., Ikeda K. (1983). Reversible blockage of membrane retrieval and endocytosis in the garland cell of the temperature-sensitive mutant of Drosophila melanogaster, shibirets1. J. Cell Biol..

[194] van der Bliek A.M., Redelmeier T.E., Damke H., Tisdale E.J., Meyerowitz E.M., Schmid S.L. (1993). Mutations in human dynamin block an intermediate stage in coated vesicle formation. J. Cell Biol..

[195] Grabs D., Slepnev V.I., Songyang Z., David C., Lynch M., Cantley L.C., De Camilli P. (1997). The SH3 domain of amphiphysin binds the proline-rich domain of dynamin at a single site that defines a new SH3 binding consensus sequence. J. Biol. Chem..

[196] Lundmark R., Carlsson S.R. (2004). Regulated membrane recruitment of dynamin-2 mediated by sorting nexin 9. J. Biol. Chem..

[197] Zoncu R., Perera R.M., Sebastian R., Nakatsu F., Chen H., Balla T., Ayala G., Toomre D., De Camilli P.V. (2007). Loss of endocytic clathrin-coated pits upon acute depletion of phosphatidylinositol 4,5-bisphosphate. Proc. Natl. Acad. Sci. USA.

[198] Bitoun M., Durieux A.C., Prudhon B., Bevilacqua J.A., Herledan A., Sakanyan V., Urtizberea A., Cartier L., Romero N.B., Guicheney P. (2009). Dynamin 2 mutations associated with human diseases impair clathrin-mediated receptor endocytosis. Hum. Mutat..

[199] Liu Y.W., Lukiyanchuk V., Schmid S.L. (2011). Common membrane trafficking defects of disease-associated dynamin 2 mutations. Traffic.

[200] Hartig S.M., Ishikura S., Hicklen R.S., Feng Y., Blanchard E.G., Voelker K.A., Pichot C.S., Grange R.W., Raphael R.M., Klip A. (2009). The F-BAR protein CIP4 promotes GLUT4 endocytosis through bidirectional interactions with N-WASp and Dynamin-2. J. Cell Sci..

[201] Kao A.W., Yang C., Pessin J.E. (2000). Functional comparison of the role of dynamin 2 splice variants on GLUT-4 endocytosis in 3T3L1 adipocytes. Am. J. Physiol. Endocrinol. Metab..

[202] Cao C., Backer J.M., Laporte J., Bedrick E.J., Wandinger-Ness A. (2008). Sequential actions of myotubularin lipid phosphatases regulate endosomal PI(3)P and growth factor receptor trafficking. Mol. Biol. Cell.

[203] Leprince C., Le Scolan E., Meunier B., Fraisier V., Brandon N., De Gunzburg J., Camonis J. (2003). Sorting nexin 4 and amphiphysin 2, a new partnership between endocytosis and intracellular trafficking. J. Cell Sci..

[204] Posey A.D., Swanson K.E., Alvarez M.G., Krishnan S., Earley J.U., Band H., Pytel P., McNally E.M., Demonbreun A.R. (2014). EHD1 mediates vesicle trafficking required for normal muscle growth and transverse tubule development. Dev. Biol..

[205] Pant S., Sharma M., Patel K., Caplan S., Carr C.M., Grant B.D. (2009). AMPH-1/Amphiphysin/Bin1 functions with RME-1/Ehd1 in endocytic recycling. Nat. Cell. Biol..

[206] Gonzalez-Jamett A.M., Momboisse F., Haro-Acuna V., Bevilacqua J.A., Caviedes P., Cardenas A.M. (2013). Dynamin-2 function and dysfunction along the secretory pathway. Front. Endocrinol..

[207] van Dam E.M., Stoorvogel W. (2002). Dynamin-dependent transferrin receptor recycling by endosome-derived clathrin-coated vesicles. Mol. Biol. Cell.

[208] Castets P., Frank S., Sinnreich M., Ruegg M.A. (2016). “Get the Balance Right”: Pathological Significance of Autophagy Perturbation in Neuromuscular Disorders. J. Neuromuscul. Dis..

[209] Klionsky D.J., Abdel-Aziz A.K., Abdelfatah S., Abdellatif M., Abdoli A., Abel S., Abeliovich H., Abildgaard M.H., Abudu Y.P., Acevedo-Arozena A. (2021). Guidelines for the use and interpretation of assays for monitoring autophagy (4th edition)(1). Autophagy.

[210] Nishino I., Fu J., Tanji K., Yamada T., Shimojo S., Koori T., Mora M., Riggs J.E., Oh S.J., Koga Y. (2000). Primary LAMP-2 deficiency causes X-linked vacuolar cardiomyopathy and myopathy (Danon disease). Nature.

[211] Masiero E., Agatea L., Mammucari C., Blaauw B., Loro E., Komatsu M., Metzger D., Reggiani C., Schiaffino S., Sandri M. (2009). Autophagy is required to maintain muscle mass. Cell Metab..

[212] Cebollero E., van der Vaart A., Reggiori F. (2012). Understanding phosphatidylinositol-3-phosphate dynamics during autophagosome biogenesis. Autophagy.

[213] Cebollero E., van der Vaart A., Zhao M., Rieter E., Klionsky D.J., Helms J.B., Reggiori F. (2012). Phosphatidylinositol-3-phosphate clearance plays a key role in autophagosome completion. Curr. Biol..

[214] Vergne I., Deretic V. (2010). The role of PI3P phosphatases in the regulation of autophagy. FEBS Lett..

[215] Schulze R.J., Weller S.G., Schroeder B., Krueger E.W., Chi S., Casey C.A., McNiven M.A. (2013). Lipid droplet breakdown requires dynamin 2 for vesiculation of autolysosomal tubules in hepatocytes. J. Cell Biol..

[216] Chen Y., Yu L. (2018). Development of Research into Autophagic Lysosome Reformation. Mol. Cells.

[217] McGrath M.J., Eramo M.J., Gurung R., Sriratana A., Gehrig S.M., Lynch G.S., Lourdes S.R., Koentgen F., Feeney S.J., Lazarou M. (2021). Defective lysosome reformation during autophagy causes skeletal muscle disease. J. Clin. Investig..

[218] East D.A., Campanella M. (2013). Ca^2+^ in quality control: An unresolved riddle critical to autophagy and mitophagy. Autophagy.

[219] Pietri-Rouxel F., Gentil C., Vassilopoulos S., Baas D., Mouisel E., Ferry A., Vignaud A., Hourde C., Marty I., Schaeffer L. (2010). DHPR alpha1S subunit controls skeletal muscle mass and morphogenesis. EMBO J..

[220] Hjerpe R., Bett J.S., Keuss M.J., Solovyova A., McWilliams T.G., Johnson C., Sahu I., Varghese J., Wood N., Wightman M. (2016). UBQLN2 Mediates Autophagy-Independent Protein Aggregate Clearance by the Proteasome. Cell.

[221] Maani N., Sabha N., Rezai K., Ramani A., Groom L., Eltayeb N., Mavandadnejad F., Pang A., Russo G., Brudno M. (2018). Tamoxifen therapy in a murine model of myotubular myopathy. Nat. Commun..

[222] Lee J.H., Lee M.J. (2012). Emerging roles of the ubiquitin-proteasome system in the steroid receptor signaling. Arch. Pharm. Res..

[223] Menconi M.J., Wei W., Yang H., Wray C.J., Hasselgren P.O. (2004). Treatment of cultured myotubes with the calcium ionophore A23187 increases proteasome activity via a CaMK II-caspase-calpain-dependent mechanism. Surgery.

[224] Robb S.A., Sewry C.A., Dowling J.J., Feng L., Cullup T., Lillis S., Abbs S., Lees M.M., Laporte J., Manzur A.Y. (2011). Impaired neuromuscular transmission and response to acetylcholinesterase inhibitors in centronuclear myopathies. Neuromuscul. Disord..

[225] Ambler M.W., Neave C., Singer D.B. (1984). X-linked recessive myotubular myopathy: II. Muscle morphology and human myogenesis. Hum. Pathol..

[226] Fidzianska A., Goebel H.H. (1994). Aberrant arrested in maturation neuromuscular junctions in centronuclear myopathy. J. Neurol. Sci..

[227] Liewluck T., Lovell T.L., Bite A.V., Engel A.G. (2010). Sporadic centronuclear myopathy with muscle pseudohypertrophy, neutropenia, and necklace fibers due to a DNM2 mutation. Neuromuscul. Disord..

[228] Lin S.S., Hsieh T.L., Liou G.G., Li T.N., Lin H.C., Chang C.W., Wu H.Y., Yao C.K., Liu Y.W. (2020). Dynamin-2 Regulates Postsynaptic Cytoskeleton Organization and Neuromuscular Junction Development. Cell Rep..

[229] Noguchi S., Fujita M., Murayama K., Kurokawa R., Nishino I. (2005). Gene expression analyses in X-linked myotubular myopathy. Neurology.

[230] Gartz Hanson M., Niswander L.A. (2015). Rectification of muscle and nerve deficits in paralyzed ryanodine receptor type 1 mutant embryos. Dev. Biol..

[231] Shakiryanova D., Klose M.K., Zhou Y., Gu T., Deitcher D.L., Atwood H.L., Hewes R.S., Levitan E.S. (2007). Presynaptic ryanodine receptor-activated calmodulin kinase II increases vesicle mobility and potentiates neuropeptide release. J. Neurosci..

[232] Relaix F., Marcelle C. (2009). Muscle stem cells. Curr. Opin. Cell Biol..

[233] Almeida C.F., Fernandes S.A., Ribeiro Junior A.F., Keith Okamoto O., Vainzof M. (2016). Muscle Satellite Cells: Exploring the Basic Biology to Rule Them. Stem Cells Int..

[234] Chazaud B. (2020). Inflammation and Skeletal Muscle Regeneration: Leave It to the Macrophages!. Trends Immunol..

[235] Tasfaout H., Cowling B.S., Laporte J. (2018). Centronuclear myopathies under attack: A plethora of therapeutic targets. J. Neuromuscul. Dis..

[236] Tasfaout H., Buono S., Guo S., Kretz C., Messaddeq N., Booten S., Greenlee S., Monia B.P., Cowling B.S., Laporte J. (2017). Antisense oligonucleotide-mediated Dnm2 knockdown prevents and reverts myotubular myopathy in mice. Nat. Commun..

[237] Trochet D., Prudhon B., Beuvin M., Peccate C., Lorain S., Julien L., Benkhelifa-Ziyyat S., Rabai A., Mamchaoui K., Ferry A. (2018). Allele-specific silencing therapy for Dynamin 2-related dominant centronuclear myopathy. EMBO Mol. Med..

[238] Childers M.K., Joubert R., Poulard K., Moal C., Grange R.W., Doering J.A., Lawlor M.W., Rider B.E., Jamet T., Daniele N. (2014). Gene therapy prolongs survival and restores function in murine and canine models of myotubular myopathy. Sci. Transl. Med..

[239] Buj-Bello A., Fougerousse F., Schwab Y., Messaddeq N., Spehner D., Pierson C.R., Durand M., Kretz C., Danos O., Douar A.M. (2008). AAV-mediated intramuscular delivery of myotubularin corrects the myotubular myopathy phenotype in targeted murine muscle and suggests a function in plasma membrane homeostasis. Hum. Mol. Genet..

[240] Mack D.L., Poulard K., Goddard M.A., Latournerie V., Snyder J.M., Grange R.W., Elverman M.R., Denard J., Veron P., Buscara L. (2017). Systemic AAV8-Mediated Gene Therapy Drives Whole-Body Correction of Myotubular Myopathy in Dogs. Mol. Ther..

[241] Lawlor M.W., Armstrong D., Viola M.G., Widrick J.J., Meng H., Grange R.W., Childers M.K., Hsu C.P., O’Callaghan M., Pierson C.R. (2013). Enzyme replacement therapy rescues weakness and improves muscle pathology in mice with X-linked myotubular myopathy. Hum. Mol. Genet..

[242] Daniele N., Moal C., Julien L., Marinello M., Jamet T., Martin S., Vignaud A., Lawlor M.W., Buj-Bello A. (2018). Intravenous Administration of a MTMR2-Encoding AAV Vector Ameliorates the Phenotype of Myotubular Myopathy in Mice. J. Neuropathol. Exp. Neurol..

[243] Raess M.A., Cowling B.S., Bertazzi D.L., Kretz C., Rinaldi B., Xuereb J.M., Kessler P., Romero N.B., Payrastre B., Friant S. (2017). Expression of the neuropathy-associated MTMR2 gene rescues MTM1-associated myopathy. Hum. Mol. Genet..

[244] Rendu J., Brocard J., Denarier E., Monnier N., Pietri-Rouxel F., Beley C., Roux-Buisson N., Gilbert-Dussardier B., Perez M.J., Romero N. (2013). Exon skipping as a therapeutic strategy applied to an RYR1 mutation with pseudo-exon inclusion causing a severe core myopathy. Hum. Gene Ther..

[245] Lim H.J., Joo S., Oh S.H., Jackson J.D., Eckman D.M., Bledsoe T.M., Pierson C.R., Childers M.K., Atala A., Yoo J.J. (2015). Syngeneic Myoblast Transplantation Improves Muscle Function in a Murine Model of X-Linked Myotubular Myopathy. Cell Transplant..

[246] Lawlor M.W., Read B.P., Edelstein R., Yang N., Pierson C.R., Stein M.J., Wermer-Colan A., Buj-Bello A., Lachey J.L., Seehra J.S. (2011). Inhibition of activin receptor type IIB increases strength and lifespan in myotubularin-deficient mice. Am. J. Pathol..

[247] Volpatti J.R., Endo Y., Knox J., Groom L., Brennan S., Noche R., Zuercher W.J., Roy P., Dirksen R.T., Dowling J.J. (2020). Identification of drug modifiers for RYR1-related myopathy using a multi-species discovery pipeline. Elife.

[248] Todd J.J., Lawal T.A., Witherspoon J.W., Chrismer I.C., Razaqyar M.S., Punjabi M., Elliott J.S., Tounkara F., Kuo A., Shelton M.O. (2020). Randomized controlled trial of N-acetylcysteine therapy for RYR1-related myopathies. Neurology.

[249] Gayi E., Neff L.A., Massana Munoz X., Ismail H.M., Sierra M., Mercier T., Decosterd L.A., Laporte J., Cowling B.S., Dorchies O.M. (2018). Tamoxifen prolongs survival and alleviates symptoms in mice with fatal X-linked myotubular myopathy. Nat. Commun..

[250] Maggi L., Mantegazza R. (2011). Treatment of myasthenia gravis: Focus on pyridostigmine. Clin. Drug Investig..

[251] Elverman M., Goddard M.A., Mack D., Snyder J.M., Lawlor M.W., Meng H., Beggs A.H., Buj-Bello A., Poulard K., Marsh A.P. (2017). Long-term effects of systemic gene therapy in a canine model of myotubular myopathy. Muscle Nerve.

[252] Shieh P.B., Bönnemann C.G., Müller-Felber W., Blaschek A., Dowling J.J., Kuntz N.L., Seferian A.M. (2020). Re: “Moving Forward After Two Deaths in a Gene Therapy Trial of Myotubular Myopathy” by Wilson and Flotte. Hum. Gene Ther..

[253] Dowling J.J., Shieh P., Kuntz N., Bonnemann C., Muller-Felber W., Lawlor M., Servais L., Smith B., Noursalehi M., Rico S. (2019). ASPIRO phase 1/2 gene therapy trial in X-linked motubular myopathy (XLMTM): Update on preliminary safety and efficacy findings. Neuromuscul. Disord..

[254] Shieh P., Kuntz N., Dowling J.J., Müller-Felber W., Blaschek A., Bönnemann C., Foley R., Saade D., Seferian A., Servais L. (2021). ASPIRO gene therapy trial in X-linked myotubular myopathy (XLMTM): Update on preliminary efficacy and safety findings. Present. WMS 2021.

[255] Laporte J., Liaubet L., Blondeau F., Tronchere H., Mandel J.L., Payrastre B. (2002). Functional redundancy in the myotubularin family. Biochem. Biophys. Res. Commun..

[256] Berger A., Maire S., Gaillard M.C., Sahel J.A., Hantraye P., Bemelmans A.P. (2016). mRNA trans-splicing in gene therapy for genetic diseases. Wiley Interdiscip. Rev. RNA.

[257] Trochet D., Prudhon B., Jollet A., Lorain S., Bitoun M. (2016). Reprogramming the Dynamin 2 mRNA by Spliceosome-mediated RNA Trans-splicing. Mol. Ther. Nucleic Acids.

[258] Rybalka E., Timpani C.A., Debruin D.A., Bagaric R.M., Campelj D.G., Hayes A. (2020). The Failed Clinical Story of Myostatin Inhibitors against Duchenne Muscular Dystrophy: Exploring the Biology behind the Battle. Cells.

[259] Koch C., Buono S., Menuet A., Robe A., Djeddi S., Kretz C., Gomez-Oca R., Depla M., Monseur A., Thielemans L. (2020). Myostatin: A Circulating Biomarker Correlating with Disease in Myotubular Myopathy Mice and Patients. Mol. Ther. Methods Clin. Dev..

[260] Mariot V., Joubert R., Hourde C., Feasson L., Hanna M., Muntoni F., Maisonobe T., Servais L., Bogni C., Le Panse R. (2017). Downregulation of myostatin pathway in neuromuscular diseases may explain challenges of anti-myostatin therapeutic approaches. Nat. Commun..

[261] Benjamin D., Colombi M., Moroni C., Hall M.N. (2011). Rapamycin passes the torch: A new generation of mTOR inhibitors. Nat. Rev. Drug Discov..

[262] Maryon E.B., Coronado R., Anderson P. (1996). unc-68 encodes a ryanodine receptor involved in regulating C. elegans body-wall muscle contraction. J. Cell Biol..

[263] Dorchies O.M., Reutenauer-Patte J., Dahmane E., Ismail H.M., Petermann O., Patthey- Vuadens O., Comyn S.A., Gayi E., Piacenza T., Handa R.J. (2013). The anticancer drug tamoxifen counteracts the pathology in a mouse model of duchenne muscular dystrophy. Am. J. Pathol..

[264] Gayi E., Neff L.A., Ismail H.M., Ruegg U.T., Scapozza L., Dorchies O.M. (2018). Repurposing the Selective Oestrogen Receptor Modulator Tamoxifen for the Treatment of Duchenne Muscular Dystrophy. Chimia.

[265] Nagy S., Hafner P., Schmidt S., Rubino-Nacht D., Schadelin S., Bieri O., Fischer D. (2019). Tamoxifen in Duchenne muscular dystrophy (TAMDMD): Study protocol for a multicenter, randomized, placebo-controlled, double-blind phase 3 trial. Trials.

